# Ultraviolet Resonant Nanogap Antennas with Rhodium Nanocube Dimers for Enhancing Protein Intrinsic Autofluorescence

**DOI:** 10.1021/acsnano.3c05008

**Published:** 2023-11-06

**Authors:** Prithu Roy, Siyuan Zhu, Jean-Benoît Claude, Jie Liu, Jérôme Wenger

**Affiliations:** † Aix Marseille Univ, CNRS, Centrale Marseille, Institut Fresnel, AMUTech, 13013 Marseille, France; ‡ Department of Chemistry, Duke University, Durham, North Carolina 27708, United States

**Keywords:** optical antennas, plasmonics, nanophotonics, ultraviolet UV, single-molecule fluorescence, tryptophan autofluorescence

## Abstract

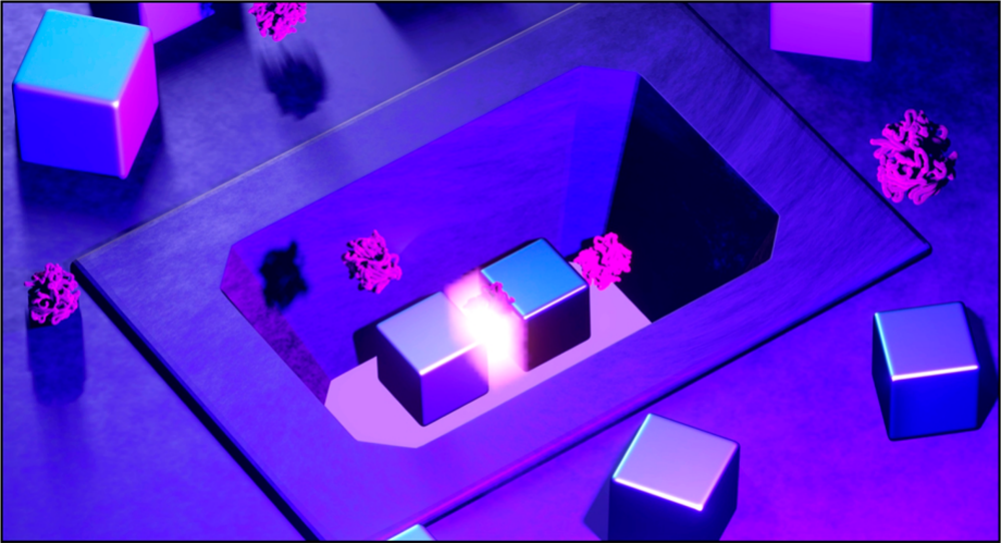

Plasmonic optical nanoantennas offer compelling solutions for enhancing light–matter interactions at the nanoscale. However, until now, their focus has been mainly limited to the visible and near-infrared regions, overlooking the immense potential of the ultraviolet (UV) range, where molecules exhibit their strongest absorption. Here, we present the realization of UV resonant nanogap antennas constructed from paired rhodium nanocubes. Rhodium emerges as a robust alternative to aluminum, offering enhanced stability in wet environments and ensuring reliable performance in the UV range. Our results showcase the nanoantenna’s ability to enhance the UV autofluorescence of label-free streptavidin and hemoglobin proteins. We achieve significant enhancements of the autofluorescence brightness per protein by up to 120-fold and reach zeptoliter detection volumes, enabling UV autofluorescence correlation spectroscopy (UV-FCS) at high concentrations of several tens of micromolar. We investigate the modulation of fluorescence photokinetic rates and report excellent agreement between the experimental results and numerical simulations. This work expands the applicability of plasmonic nanoantennas to the deep UV range, unlocking the investigation of label-free proteins at physiological concentrations.

## Introduction

The interaction between light and a single fluorescent molecule is fundamentally limited by the over 100-fold size mismatch between their respective wavelengths and dimensions,^1^ leading to a weak net fluorescence signal per molecule in diffraction-limited microscopes. As a result, the sensitivity and temporal resolution of single-molecule fluorescence techniques, which are essential in modern biophysics and biochemistry, are also limited.^2,3^ To overcome these limits, plasmonic optical nanoantennas have been introduced to manipulate light at the deeply subwavelength scale and enhance the light–matter interactions.^4,5^ A broad range of optical nanoantenna designs including bowtie,^6,7^ single nanorod,^8−10^ nanoparticle on mirror,^11−13^ nanoparticle assemblies,^14^ DNA-origami dimer,^15−21^ DNA-templated dimer,^22−24^ metasurface,^25−27^ or antenna-in-box^28,29^ has been demonstrated to significantly enhance the fluorescence brightness of single molecules, reaching impressive fluorescence enhancement factors above 1000-fold. By manipulating light at the nanoscale, the optical nanoantennas provide exquisite control over the radiation properties of a single quantum emitter, providing various possibilities to tune the directionality of the fluorescence light,^30,31^ achieve ultrafast photoemission,^11,12^ reduce the photobleaching rate,^32−34^ control the near-field dipole–dipole energy transfer,^35,36^ or trap single nano-objects.^37,38^

However, the current demonstrations and operating ranges of nanoantennas remain largely limited to the visible and near-infrared regions. While this range is well suited for organic fluorophores and quantum dots, extending it toward the ultraviolet (UV) region brings the key additional benefit of exploiting directly the autofluorescence of proteins without requiring any additional fluorescent label.^39−47^ Over 90% of all human proteins contain tryptophan or tyrosine amino acid residues which are naturally fluorescent in the UV.^40,48^ Exploiting this intrinsic UV autofluorescence signal is an appealing route to monitor single label-free proteins, releasing the need for any external fluorescence labeling.^49^ The issues related to external fluorescence labeling are not only a matter of time and cost of preparation but also mainly the potential adverse effects the fluorescent marker may have on the protein conformation and/or dynamics, as documented by several reports.^50−59^

Despite the growing interest in utilizing the UV range to enhance the light–matter interaction, there have been limited reports about UV resonant optical nanoantennas.^60^ Earlier works concerned mostly aluminum nanoparticle arrays to enhance Raman scattering^61−66^ and fluorescence^67−72^ from dense molecular layers. Another important class of UV nanoantennas is subwavelength nanoapertures,^73−79^ which can be combined with a microreflector to increase the collection efficiency.^48,80^ However, all these designs are only weakly resonant and lack the strong field confinement achieved with gap surface plasmon resonances.^1^ Although numerical investigations have explored UV resonant nanoparticles^81,82^ and dimer gap antennas^83−87^ to achieve higher local field enhancement, their experimental demonstrations have been limited so far to Raman scattering^88^ and near-field imaging.^89^ The major application of enhancing the autofluorescence of label-free proteins remains unexplored. Beyond the challenging difficulty of such experiments, another limiting factor is the poor stability of aluminum plasmonics structures in an aqueous environment,^90−93^ especially under UV illumination.^94,95^ While coating with silica or other oxide materials can promote the Al corrosion stability,^93,95^ this comes at the expense of an ∼10 nm thick supplementary layer which in turn enlarges the gap size and reduces the net enhancement in the antenna hot spot.

**Figure 1:**
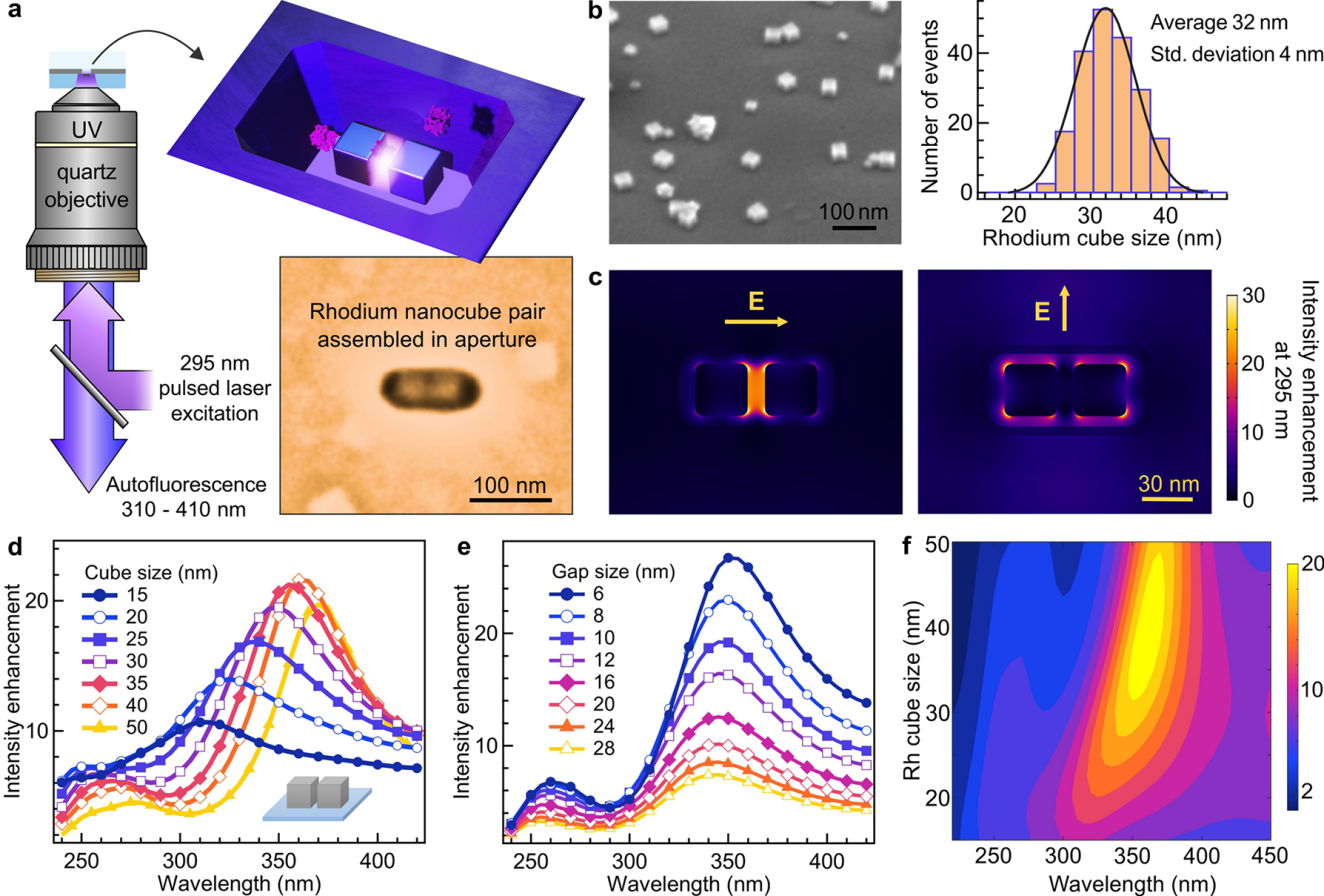
UV nanogap antenna assembled with rhodium nanocubes. (a) Scheme of the UV microscope with the resonant nanogap antenna made from two 30 nm rhodium nanocubes assembled into a 120 × 50 nm rectangular aperture milled in an opaque aluminum film. The inset shows a scanning electron microscope (SEM) image of a nanoantenna. (b) SEM image of rhodium nanocubes dispersed on an ITO-coated coverslip viewed at 52° incidence. The size distribution corresponds to the length of the rhodium nanocubes as measured by SEM, without any deconvolution or data treatment. (c) Numerical simulations of the electric field intensity enhancement at 295 nm for a UV nanogap antenna made of two 30 nm rhodium cubes separated by a 10 nm gap in a rectangular nanoaperture milled into an aluminum film. The arrows indicate the orientation of the incident electric field. The device is immersed in water. (d–f) Numerical simulations of the spectral dependence of the intensity enhancement in the center of the nanogap antenna as a function of the rhodium nanocube size and gap size. For (d) and (f) the gap size is set at 10 nm, while for (e) the cube size is 30 nm. To speed up the numerical calculations and provide design guidelines, the simulations in (d–f) consider only a pair of rhodium nanocubes on a quartz coverslip immersed in water; there is no aluminum layer here.

Here we simulate, fabricate, and characterize UV resonant nanogap antennas made of dimers of rhodium nanocubes to enhance the tryptophan autofluorescence from label-free proteins. Rhodium nanoantennas provide a powerful solution to the water corrosion issue associated with aluminum,^96,97^ while maintaining a good plasmonic response down to the deep UV range.^81^ Our fabrication approach relies on capillary-assisted self-assembly of rhodium nanocubes into rectangular nanoholes milled into a quartz substrate,^98,99^ leaving the nanogap region free of organic molecules for detecting diffusing proteins over a minimal luminescence background (Figure 1a and Figure S1). Our UV resonant nanogap antennas provide enhancement factors up to 120-fold for the autofluorescence brightness of single proteins. The UV light is concentrated into 40 zL detection volumes, which in turn enables UV autofluorescence correlation spectroscopy (UV-FCS) at concentrations exceeding 50 μM.^100,101^ We also investigate the modification of different fluorescence photokinetic rates by rhodium nanoantennas and demonstrate excellent agreement between our experiments and numerical simulations. Overall, our study expands the applicability of plasmonic nanoantennas down to the deep UV range,^4,5^ broadening the capabilities to interrogate single proteins in their native state at physiological concentrations.^100−102^

## Results and Discussion

The synthesis of the rhodium nanocubes follows the protocol published in the literature^96^ based on slow injection of polyols. This approach allows precise tunability of the nanocube size as well as a narrow size distribution. The key advantages of rhodium in this context are (i) the precise control of the nanocube size and shape, allowing the tuning of the plasmonic resonance down into the UV region, (ii) the resistance to UV-induced photocorrosion largely outperforming aluminum,^94,95^ and (iii) the absence of a native oxide layer to maximize the nanogap enhancement. Here, we selected a nanocube size of around 30 nm (Figure 1b). As we discuss below, this size ensures that the plasmonic resonance of the dimer antenna occurs near 350 nm, which matches with the peak autofluorescence emission of tryptophan.^73^

The fabrication of the dimer nanogap antennas relies on the capillary-assisted self-assembly applied to the rhodium nanocubes.^98,99^ A focused ion beam (FIB) is used to mill rectangular nanoapertures into an aluminum-covered quartz substrate to serve as a template for the nanocube self-assembly (see Materials and Methods for complete experimental details). The 120 × 50 nm^2^ size of the nanoaperture is chosen to accommodate only two nanocubes and leave an ∼10 nm gap between them (Figures S1 and S2). Figure 1a shows a typical scanning electron microscope (SEM) image of an assembled nanogap antenna, with more examples provided in Figure S1 of the Supporting Information. Correlative measurements between the SEM and the UV microscope using fiducials on the sample allow selection of only the antennas where a dimer of rhodium nanocubes is clearly seen (Figure S3). Importantly in this study, Figure S1 shows the SEM image for each nanoantenna probed in the UV microscope. Each antenna is identified with an alphanumeric code together with a specific symbol, allowing correlation of the specific geometry of the nanoantenna with its optical performance. We meticulously evaluate the gap sizes for each SEM image (Figure S1) and consistently achieve gap dimensions ranging between 10 and 20 nm, with a median gap size of 14 nm. These results exhibit favorable comparisons with top-down fabrication methodologies, such as focused ion beam and electron-beam lithography, as illustrated in Figure S2. However, it is important to note that the primary focus of our research is not centered on developing a nanofabrication technique. Instead, our key objective is to showcase the successful realization of ultraviolet nanogap antennas and demonstrate their performance for detecting label-free proteins. As additional advantages of our design, we benefit from the single crystallinity of the rhodium nanocubes to reduce the plasmonic losses.^1,9^ The aluminum layer serves to block the direct illumination of the molecules diffusing away from the nanoantenna but still present in the confocal volume, as with the antenna-in-box design (Figure 1a).^28,29^ This method also leaves the nanogap region completely free of organic molecules, which is important to reduce the residual UV luminescence background for the detection of diffusing proteins.

Numerical simulations based on the finite element method confirm the excitation of resonant nanogap modes when the excitation polarization is set parallel to the dimer’s main axis. Figure 1c shows the intensity maps for 295 nm excitation, which was used in our experiments on proteins, as this wavelength gives a slightly better signal to background ratio than the 266 nm laser line. The intensity maps for 266 nm (*p*-terphenyl excitation) and 350 nm (peak autofluorescence emission) are shown in Figures S4 and S5 in the Supporting Information, respectively, with an intensity profile similar to that found for 295 nm (Figure 1c). Even in cases of significant misalignment of the nanocubes, substantial optical confinement and intensity enhancement are still predicted by numerical simulations (Figure S6).

Increasing the size of the nanocube leads to a red shift of the plasmonic resonance (Figure 1d). Reducing the gap size increases the intensity enhancement in the nanogap region and also leads to a red shift of the resonance (Figure 1e). Altogether, these features demonstrate the occurrence of plasmonic nanogap resonances in rhodium dimer antennas.^4,5^ Using a parametric study as a function of cube size, gap size, and resonance wavelength (Figure 1f and Figure S7), we select nanocube sizes of around 30 nm for the autofluorescence enhancement experiments. This leads to a plasmonic resonance slightly blue-shifted respective to the peak protein emission wavelength at 350 nm (Figure S8), as this condition has been proven to yield the best brightness enhancement factors.^103^

**Figure 2:**
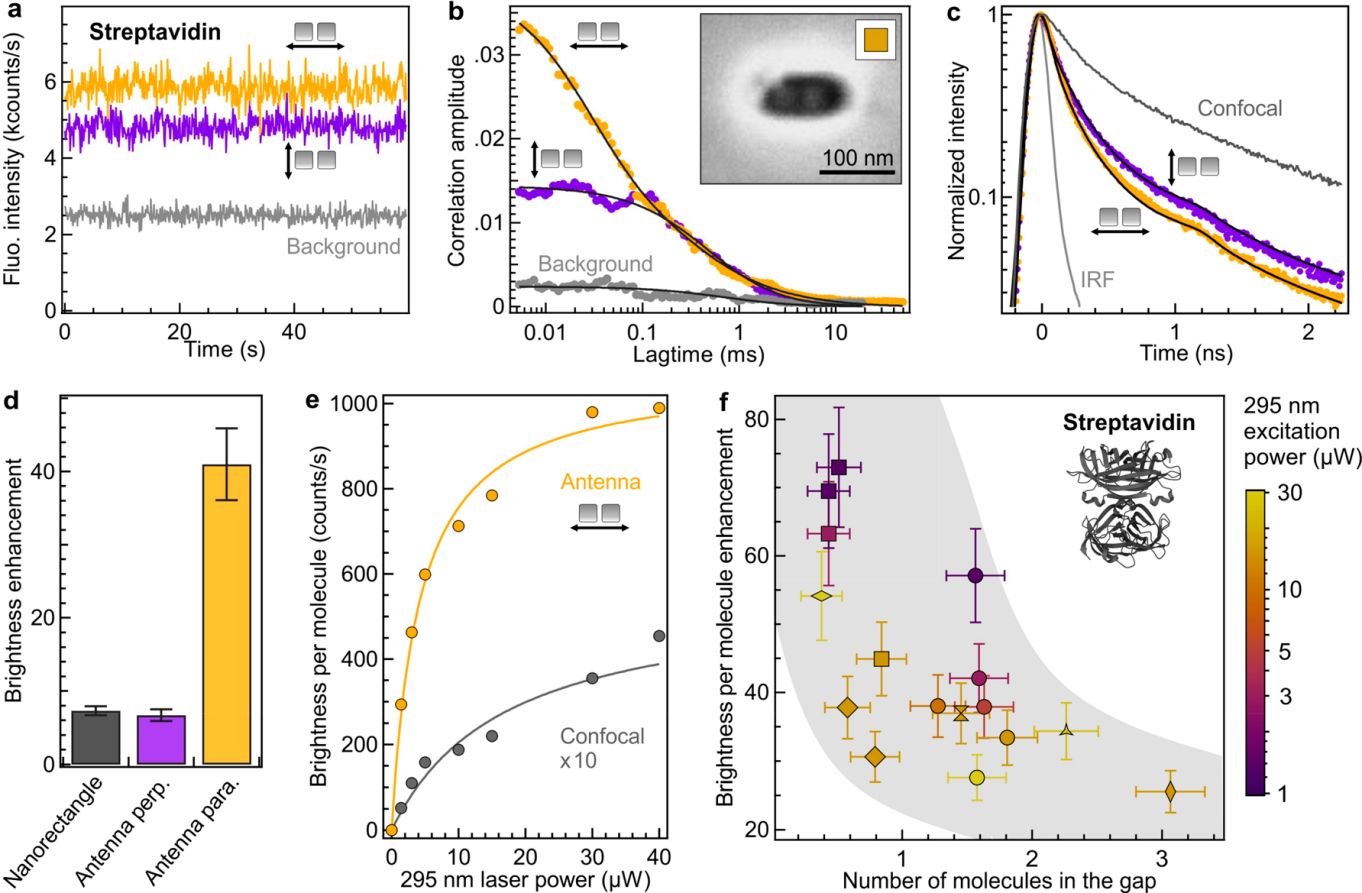
UV autofluorescence from label-free streptavidin proteins enhanced with a nanogap antenna. (a) Autofluorescence intensity time traces (binning time of 100 ms) for a rhodium nanogap antenna with the excitation polarization set parallel (yellow) or perpendicular (purple) to the dimer antenna’s main axis. The antenna is covered with a 50 μM solution of diffusing label-free streptavidin proteins. The gray trace shows the background intensity level in the absence of the protein (the antenna is covered with the buffer solution). The 295 nm excitation power used here is 15 μW. (b) FCS correlation functions corresponding to the traces in (a). Dots are experimental data, and lines are numerical fits. The insert SEM image shows the dimer antenna used for this experiment (the antenna reference is R5s1p5e2-1 with a gap size of 10 nm as measured by SEM; see Figure S1). The data corresponding to this antenna appear as square markers in the scatter plot (f). (c) Normalized time-resolved decay traces corresponding to the experimental data in (a) and to the confocal reference (dark gray). IRF stands for the instrument response function. (d) Comparison of the enhancement factors for the fluorescence brightness per molecule in the empty nanorectangle (without rhodium nanocubes, see Figure S9) and the rhodium nanoantenna with parallel and perpendicular excitation polarizations. (e) Excitation power dependence of the brightness per molecule measured in the nanogap antenna (yellow markers) and in the confocal reference (gray). The line is a fit with a saturation model.^28^ (f) Scatter plot of the fluorescence brightness enhancement for streptavidin proteins as a function of the number of detected molecules in the gap antenna. Different markers indicate different nanogap antennas, whose SEM images are shown in Figure S1. The color codes indicate the excitation power, and the shaded area is a guide for the eyes.

We used fluorescence correlation spectroscopy (FCS) and time-correlated single photon counting (TCSPC) experiments to assess the optical performance of the nanoantennas and their ability to enhance the UV autofluorescence of diffusing label-free proteins. The comparison between experiments performed with the excitation laser polarization set parallel and perpendicular to the main antenna axis demonstrates the contribution of nanogap enhancement. Figure 2 summarizes the results found with label-free streptavidin at a 50 μM concentration. A higher intensity is obtained when the excitation polarization is set parallel to the gap (Figure 2a). We have checked that the excitation and detection on our microscope are not polarization sensitive, so that the polarization dependence can be directly linked with the enhanced autofluorescence signal stemming from the nanoantenna gap region. However, relying solely on the total intensity averaged across the entire antenna volume is inadequate for estimating the brightness enhancement per molecule. This is because the total intensity comprises the product of brightness and the number of molecules. To overcome this challenge, we employ FCS as a powerful technique to independently determine both the number of molecules contributing to the signal and their individual autofluorescence brightness per emitter.^14,28,29^ In addition, FCS is supplemented with time-correlated single photon counting (TCSPC) to estimate the fluorescence lifetime.

Looking at the raw data, we readily observe that the FCS curve has a higher correlation amplitude with parallel excitation polarization than with perpendicular orientation (Figure 2b and Table S1), while the autofluorescence lifetime is reduced when the excitation polarization is turned from perpendicular to parallel (Figure 2c and Table S2). The nanoantenna significantly reduces the autofluorescence lifetime from 1.5 ns for the confocal reference to 0.47 ns for the antenna with parallel orientation, demonstrating a higher local density of optical states (LDOS) in the nanoantenna hot spot and Purcell effect on label-free proteins.^1^ All of these raw observations highlight the contribution of the nanogap hot spot and its effect to enhance the UV autofluorescence.

To quantify the brightness enhancement with the nanoantenna, we used UV-FCS to measure the average number of molecules N* present inside the nanogap and their autofluorescence brightness per molecule Q* (see Materials and Methods). This general FCS approach has been validated previously for plasmonic antennas in the visible and organic fluorescent dyes.^14,28,29,35^ For the nanoantenna with parallel excitation, we find a brightness enhancement of 41 ± 5-fold for label-free streptavidin (Figure 2d). This performance is clearly above the enhancement found with the perpendicular orientation (6.7 ± 0.8) or the empty nanoaperture in the absence of rhodium antenna (7.3 ± 0.6, see Figure S9). We also perform experiments on single rhodium nanocubes (Figure S10) which yield enhancement values similar to those of the empty nanoaperture, confirming the specific optical response from the nanogap with parallel excitation. Importantly, the enhancement factor found with the nanoantenna and parallel polarization significantly outperforms the gain obtained earlier with nanoaperture-based designs (we obtained 4-fold enhancement^78^ for a bare nanoaperture without a microreflector and 15-fold with the so-called horn antenna^80^ combining a nanoaperture and a microreflector). This superior performance is achieved by combining UV plasmonic resonant gap modes, yielding intense electromagnetic enhancements together with the corrosion-resistance and single-crystalline nature of the rhodium nanocubes.

The observation of saturation of the autofluorescence brightness (Figure 2e) is a supplementary control to show that the signal stems from protein autofluorescence and is not related to some laser backscattering or Raman scattering. Streptavidin autofluorescence brightness up to 1000 photons/(s molecule) are reached, which is a key element in maximizing the signal-to-noise ratio in UV-FCS.^48^ Deep UV nanoantennas offer a transformative opportunity to substantially amplify the autofluorescence signal from individual label-free proteins and thus render previously undetectable signals easily discernible. Leveraging UV-FCS experiments unlocks powerful perspectives to assess local concentrations, mobilities, brightness, and stoichiometries of label-free proteins.

For FIB^28^ as well as for electron-beam lithography,^29^ some variability in the nanoantenna gap size (Figure S2) inevitably leads to a dispersion of the nanoantenna performance. We assess this effect for our UV antennas with Figure 2f displaying the brightness enhancement as a function of the number of gap molecules N*. Importantly here, each data point in Figure 2f can be assigned to a specific SEM image of the antenna. As found earlier for visible antennas and fluorescent dyes,^29^ there is a correlation between the brightness enhancement and the number of detected molecules in the gap, with antennas having a narrower gap tending to give a higher brightness enhancement and a lower number of molecules. We do monitor a similar feature here for UV antennas and label-free proteins. We also find a clear correlation between the volume measured with FCS and the gap size obtained from the SEM images (Figure S11). This allows us to retrieve the evolution of the brightness enhancement as a function of the gap size (Figure S12), where we find that the antennas with the smallest gaps provide the highest brightness enhancements.

With an average number of molecules in the nanogap of 1.3 ± 0.7 for a 50 μM concentration, the nanoantenna detection volume thus corresponds to 40 ± 20 zL (1 zL = 10^–21^ L = 1000 nm^3^), 25000-fold below the femtoliter confocal detection volume. For comparison, experiments on gold nanoantennas with 12 nm gaps led to detection volumes of 100 zL, while dimers of spherical 80 nm gold nanoparticles gave volumes of 70 zL.^14^ Our correlative UV-FCS and SEM measurements clearly underscore the FCS volume dependence on the gap size (Figure S11a) with the smallest 10 nm gaps yielding volumes down to 15 zL while the largest 26 nm gaps providing volumes around 100 zL.

**Figure 3:**
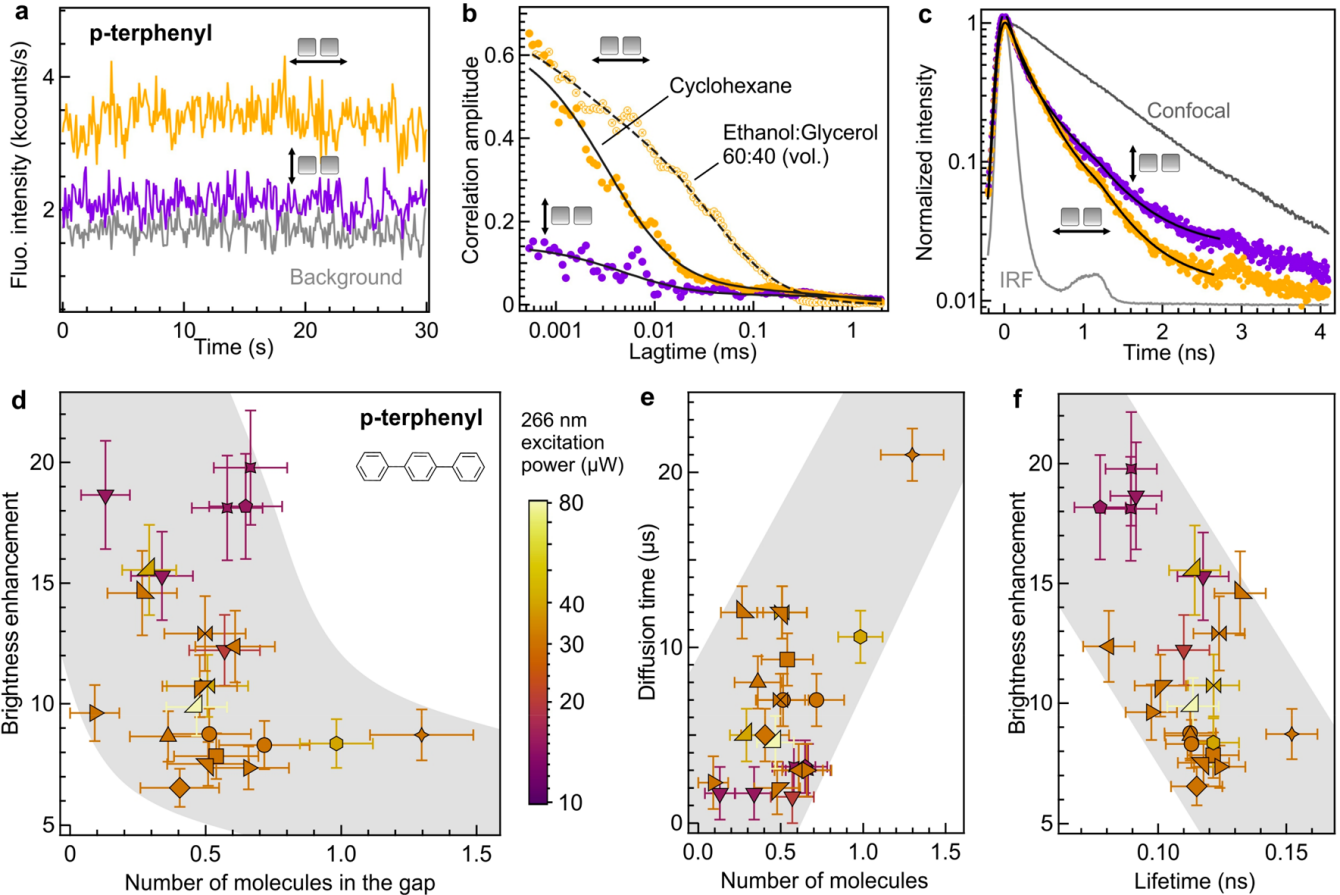
UV fluorescence enhancement of *p*-terphenyl with rhodium nanogap antennas. (a) Fluorescence intensity time traces recorded on a 10 μM solution of *p*-terphenyl in cyclohexane on a nanoantenna with the excitation polarization set parallel (yellow) or perpendicular (purple) to the dimer antenna’s main axis. The bonding time is 100 ms. The gray trace shows the background intensity level in the absence of *p*-terphenyl. The 266 nm excitation power used here is 40 μW. The data in (a–c) correspond to the antenna reference number R10s1p2d5-8 for which a 11 nm gap size was inferred from the SEM image (Figure S1). (b) FCS correlation functions corresponding to the traces in (a) and when *p*-terphenyl molecules are diluted into a 60/40 glycerol/ethanol mixture to increase the viscosity. Dots are experimental data, and lines are numerical fits. (c) Normalized time-resolved decay traces corresponding to the experimental data in (a) and to the confocal reference (dark gray). (d) Scatter plot of the fluorescence brightness enhancement for *p*-terphenyl as a function of the number of molecules detected in the gap antenna. The various markers indicate different nanoantennas, whose SEM images are shown in Figure S1. The color codes indicate the excitation power. Among the different experiments, the number of molecules has been scaled to correspond to a 30 μM concentration of *p*-terphenyl. (e) Scatter plot of the FCS diffusion time as a function of the number of molecules detected in the nanogap. (f) Scatter plot of the fluorescence brightness enhancement as a function of the fluorescence lifetime. Throughout parts (d–f), the shaded areas are guides to the eyes.

The UV-FCS experiments are repeated with *p*-terphenyl (Figure 3) and hemoglobin (Figure S13) to probe a wider range of conditions and initial quantum yield of the emitters. *p*-terphenyl is a UV fluorescent dye with 93% quantum efficiency,^73^ while our comparative spectroscopy experiments estimate the average quantum yield of hemoglobin to be around 0.5% (Figure S8). The characteristic polarization-dependent signature of the dimer gap antenna is observed again for both *p*-terphenyl (Figure 3a–c) and hemoglobin (Figure S13). Experiments with *p*-terphenyl are further used to control the diffusion time across the nanoantenna scaling with the solution viscosity. While replacing cyclohexane by a 60/40 (v/v) glycerol/ethanol mixture, confocal experiments show that the solution viscosity increases by 12.5-fold. Our nanoantenna data (Figure 3b) retrieve a similar increase of 11.4-fold of the diffusion time which we directly relate to the increase in the solution viscosity.

Looking at the statistics from 27 antennas, we note that smaller gap volumes lead to higher brightness enhancement factors (Figure 3d and Figure S12) and shorter diffusion times (Figure 3e). We relate both features to a better confinement of light into nanoscale dimensions, as demonstrated by the SEM gap sizes (Figures S1 and S11). The detection volume inferred from UV-FCS on *p*-terphenyl is 30 ± 15 zL, which stands in good agreement with the streptavidin data. Our data also display the interesting trend that the brightness enhancement scales inversely with the fluorescence lifetime (Figure 3f). This indicates that in the range of conditions probed here, the antennas with smaller gaps lead to higher molecular brightness and higher LDOS (shorter lifetime), as expected for resonant plasmonic nanoantennas.

**Figure 4:**
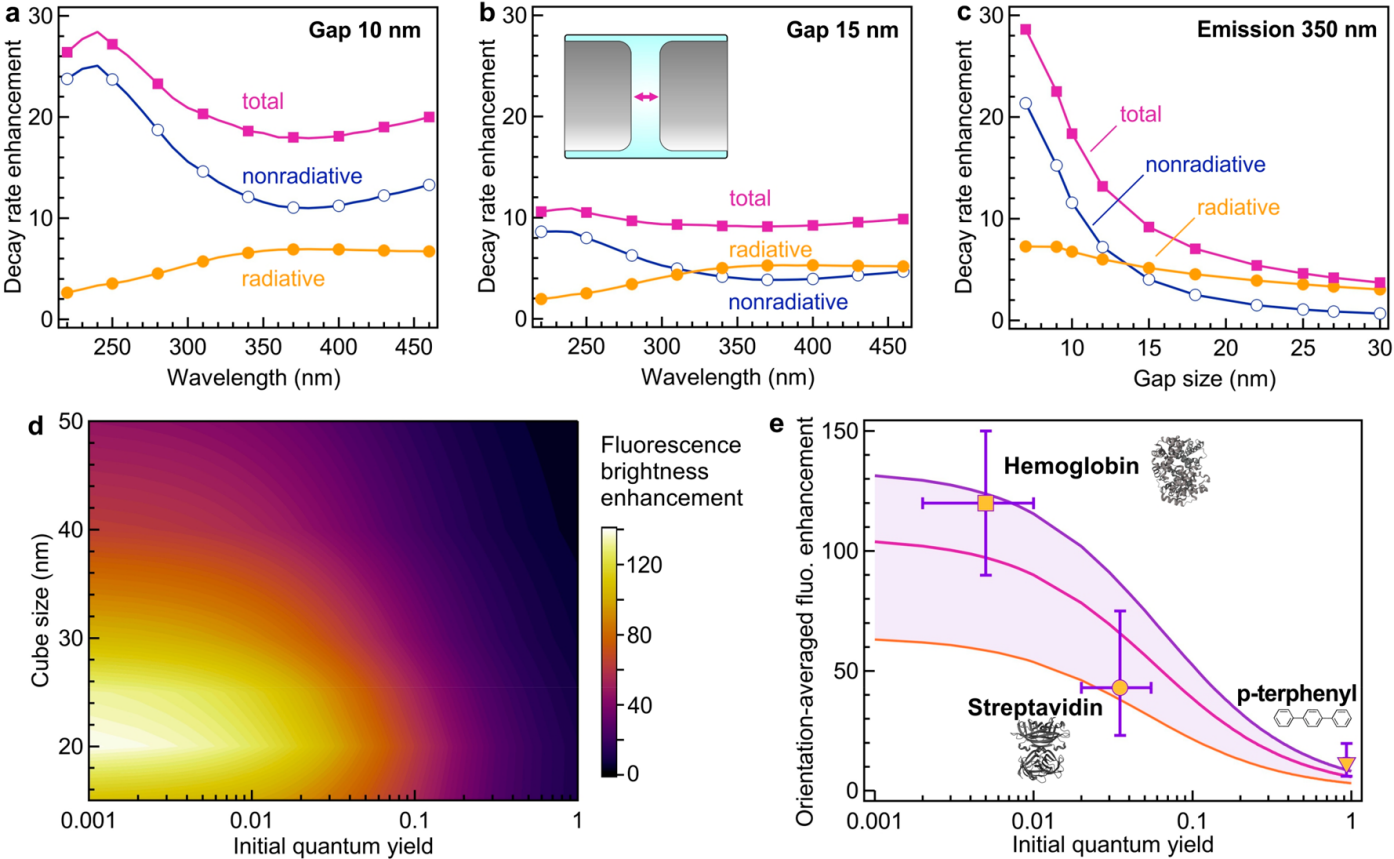
Rhodium UV nanogap antennas to enhance the photokinetic rates. (a, b) Numerical simulations of the enhancement of the decay rate constants as a function of the emission wavelength for a perfect dipole emitter with parallel orientation located in the center of the gap between two rhodium nanocubes in water. The rhodium cube size is constant at 30 nm. The gap size is 10 nm in (a) and 15 nm in (b). All rates are normalized, respectively, to the dipole radiative rate in free space. (c) Evolution of the decay rate enhancement factors as a function of the gap size, for an emission wavelength of 350 nm and a cube size of 30 nm. (d) Simulations of the fluorescence brightness enhancement as a function of the rhodium nanocube size and the emitter’s initial quantum yield in free space. The gap size is kept constant at 10 nm. The excitation wavelength is 295 nm, and the emission is 350 nm. The emission is averaged over the three orientation directions. (e) Comparison of the simulated (lines) and experimental (markers) fluorescence brightness enhancement factors as a function of the quantum yield in a homogeneous solution for the different emitters used in this work. The emission is averaged over the three orientation directions. From top to bottom, the three lines represent rhodium cube sizes of 25, 30, and 40 nm, respectively, with a gap size set to 10 nm. The protein structures in gray have been made using Mol* viewer.^104^

To better understand the physics behind the UV fluorescence enhancement and assess the influence of plasmonic losses, we perform numerical simulations and estimate the antenna’s influence on the radiative, nonradiative, and total decay rate constants (Figure 4a–c and Figures S14 and S15). Nanoantennas made of 30 nm rhodium cubes have a dipolar resonance around 350 nm and a quadrupolar resonance around 260 nm. The quadrupolar resonance is essentially nonradiative. This effect, together with the increased intrinsic losses of rhodium below 300 nm, explains the increase of the nonradiative rate enhancement and the drop of the antenna efficiency below 300 nm (Figure S14). The different decay rate constants critically depend on the gap size (Figure 4c), with the smallest gap sizes below 10 nm being dominated by nonradiative losses.

With the knowledge of the excitation intensity gain (Figure 1c) and the antenna’s influence on the various photokinetic rates, we can infer the net fluorescence brightness enhancement as being a function of the emitter’s initial quantum yield^17,29^ and compare with our experimental results. Emitters with lower quantum yields give higher apparent brightness enhancement factors (Figure 4d) as a maximum benefit can be taken from the nanoantenna’s ability to enhance the radiative rate.^17,29^ We compare our experimental results to the numerical predictions in Figure 4e. Within the experimental uncertainties, the enhancement values found for the different molecules agree well with the theoretical predictions, confirming the validity of our approach. Experimentally, the highest brightness enhancement of 120-fold is obtained with hemoglobin, which has the lowest 0.5% quantum yield in solution. The simulations predict even higher enhancement factors above 400-fold, yet in the case of a dipolar source perfectly aligned with the nanoantenna (Figure S16).

Combining all of the experimental results on the brightness enhancement and the fluorescence lifetime reduction, we can compute back all of the different decay rate constants (Table S3 in the Supporting Information). For *p*-terphenyl, streptavidin, and hemoglobin, despite the large difference in their initial quantum yields, we find consistent excitation gains η_exc_ = 15.5 ± 3.8 and radiative gains η_Γrad_ = 10.8 ± 2.6 in good agreement with numerical simulations considering the experimental values are orientation-averaged and position-averaged inside the nanogap. The loss decay rate constant into the metal is also a preserved feature among our different experiments, with Γ_loss_^*^ = 1.25 ± 0.3 ns^–1^. Comparing with nanogap antennas in the red spectral range with comparable gap sizes, a loss decay rate constant of 0.5 ns^–1^ can be found for gold,^16,23^ while aluminum and silicon yield typically 2 and 4 ns^–1^, respectively.^35,105^ The nonradiative losses associated with rhodium in the UV thus appear quite comparable to other materials in the visible range.

In the future, aluminum nanocubes^106^ and nanocrystals^93^ could allow reaching lower losses than for rhodium, provided their water corrosion issue can be circumvented. We have performed numerical simulations to assess the UV performance of optimized aluminum nanoantennas and compare them with the rhodium antennas discussed in this work. Figure S17 in the Supporting Information summarizes our main results. We find that pure aluminum outperforms rhodium by approximately 50%, aligning with our initial expectations. However, the introduction of a supplementary oxide layer to safeguard aluminum against UV photocorrosion leads to a notable drop in performance, with the enhancement being 3 times lower for protected aluminum as compared to that of pure rhodium. These compelling results further underscore the interest for rhodium in UV plasmonic applications and the need for specific care while designing protective measures for the nanoantennas.

## Conclusions

In conclusion, our work provides experimental and numerical evidence for the successful implementation of rhodium nanogap antennas with plasmonic resonances extending deep into the ultraviolet region. By harnessing the combination of intense electric field enhancement and photokinetic rate alteration, our antenna design achieves brightness enhancement factors up to 120-fold, together with detection volumes in the zeptoliter range and subnanosecond autofluorescence lifetime. Notably, correlative SEM and UV-FCS measurements demonstrate that the nanogap mode plays a pivotal role, as is evident from polarization-dependent measurements and the interdependence observed among brightness, lifetime, and detection volume. Thanks to the intense nanogap enhancement, the plasmonic resonant nature of the gap mode, and the single crystallinity and smooth surface of the rhodium nanocubes, the optical performance of our antennas significantly outperforms previous nanoparticle- or nanoaperture-based devices.

Enhancing the autofluorescence of label-free proteins stands as a major application and driving motivation for UV plasmonics. To showcase this capability, we present the successful enhancement of the autofluorescence signals from streptavidin and hemoglobin proteins. While the autofluorescence quantum yield of tryptophan in most proteins is typically on the order of a few percent,^47^ there is a compelling interest in utilizing UV nanoantennas to significantly amplify the autofluorescence signal from single proteins, rendering it easily detectable. Leveraging UV-FCS experiments, we unlock powerful perspectives for local measurements of concentration, mobility, brightness, and stoichiometry of label-free proteins.^100−102^

Altogether, our work significantly advances the field of nanotechnology and biosensing by demonstrating the success of ultraviolet nanogap antennas for label-free protein detection. Extending the practical application of plasmonic nanoantennas into the deep UV range broadens the capabilities to investigate individual proteins in their native state under physiological concentrations.^100−102^ The robustness of the achieved gap sizes further enhances the practical applicability of our approach, positioning it as a promising technique in this domain. Beyond label-free protein autofluorescence detection, resonant UV nanoantennas are highly relevant to advance several other plasmonic applications, including resonant Raman spectroscopy,^61,79^ circular dichroism spectroscopy,^107^ photodetectors,^108^ and photocatalysis.^109^

## Materials and Methods

### Rhodium Nanocube Synthesis

Rhodium nanocubes were synthesized using a seed-mediated method reported earlier.^96^ First, a rhodium seed solution was prepared: 0.45 mmol of KBr (ACROS, reagent ACS) was dissolved in 2 mL of ethylene glycol (J.T. Baker, 99.0%) in a 20 mL scintillation vial. The vial was put in an oil bath for 40 min at 160 °C. Subsequently, 0.045 mmol of RhCl_3_·*x*H_2_O (Aldrich, 98%) and 0.225 mmol of PVP (Aldrich, mw = 55000) were dissolved in 2 mL of ethylene glycol separately. A two-channel syringe pump was used to pump these two solutions into the vial at a speed of 1 mL/h. The solution was aged for 10 min at 160 °C before cooling to room temperature as the seed solution. 0.4 mL of the prepared rhodium seed solution was then mixed with 1.6 mL of ethylene glycol for a total of 2 mL in another 20 mL scintillation vial. The vial was put in an oil bath for 40 min at 160 °C. Two additional solutions produced by dissolving 0.045 mL of RhCl_3_·*x*H_2_O in 2 mL of ethylene glycol and 0.225 mmol of PVP (Aldrich, mw = 55000) plus 0.45 mmol of KBr were in another 2 mL of ethylene glycol. These two solutions were pumped into the heated vial at a speed of 1 mL/h. After all solutions were added, the mixture was cooled to room temperature, and rhodium nanocubes were collected after centrifugation and washed with water/acetone several times.

### Nanoantenna Fabrication

Arrays of 120 nm × 50 nm rectangular nanoapertures together with fiducial marks were milled by a focused ion beam (FIB) on a UV-transparent quartz coverslip substrate covered with a 100 nm thick aluminum layer. FIB milling was performed on an FEI DB235 Strata instrument with 30 kV acceleration voltage and 10 pA gallium ion current. The aluminum nanoapertures were covered by a 10 nm thick silica layer deposited with plasma-enhanced chemical vapor protection (Oxford Instruments PlasmaPro NGP80) in order to protect the aluminum layer against UV-induced photocorrosion.^94,95^ For the deposition of rhodium nanocubes and their self-assembly into nanogap antennas, 2 mM sodium dodecyl sulfate (SDS) and 1% Tween20 were added to 100 μL of the rhodium solution. This solution was then left for 15 min in an ultrasonic bath to ensure all nanoparticles were well dispersed. Droplets of 4 μL were then deposited on the aluminum nanorectangle sample and left to evaporate. Different movements of the droplet, respective to the sample, have been tried to benefit from capillary-assisted self-assembly, but the simple horizontal evaporation of the rhodium nanocube droplet gave the best results in our case. As we were illuminating from below the quartz substrate, the presence of extra nanoparticles on top of the aluminum film had no effect on our measurements. The nanoantennas were then imaged with a scanning electron microscope (electron beam of the FEI DB235 Strata). The position of the antennas containing two rhodium nanocubes was noted and used later to find the same antennas in the UV microscope. The rhodium antennas were remarkably stable; the sample could be rinsed and dried several times without disturbing the antenna geometry.

### Fluorescent Samples

*p*-Terphenyl, *Streptomyces avidinii* streptavidin, and human hemoglobin were purchased from Sigma-Aldrich in powder form (see complete details in Table S4). *p*-Terphenyl was dissolved in cyclohexane, while the proteins were dissolved in a 25 mM Hepes, 300 mM NaCl, 0.1 v/v% Tween20, 1 mM DTT, and 1 mM EDTA 1 mM buffer solution at pH 6.9. The solutions were centrifuged for 12 min at 142000*g* (Airfuge 20 psi), and the supernatants were stored at −20 °C and further used for the experiments. The concentrations were assessed with a Tecan Spark 10 M fluorometer. Prior to the UV measurements, the oxygen dissolved in the solution was removed by bubbling the buffer with argon for 5 min, and 10 mM of mercaptoethylamine MEA was added to improve the photostability.

### UV Microscopy

For protein experiments, we used a 295 nm laser (Picoquant Vis-UV-295-590) while for *p*-terphenyl we used a 266 nm laser (Picoquant LDH-P-FA-266). Both lasers were pulsed with a 70 ps duration and 80 MHz repetition rate. The laser beams were spatially filtered by a 50 μm pinhole to achieve quasi-Gaussian beam profiles. The UV microscope objective was a LOMO 58× 0.8 NA with water immersion. The nanoantenna sample was scanned by a three-axis piezoelectric stage (Physik Instrumente P-517.3CD). The fluorescence light was collected by the same microscope objective, separated from the excitation laser beam by a dichroic mirror (Semrock FF310-Di01-25-D) and focused onto a 50 μm pinhole by a quartz lens with 200 mm focal length (Thorlabs ACA254-200-UV). Emission filters (Semrock FF01-300/LP-25 and FF01-375/110–25) were placed before the photomultiplier tube (Picoquant PMA 175), whose output was connected to a time-correlated single photon counting TCSPC module (Picoquant Picoharp 300 with time-tagged time-resolved mode). The full width at half-maximum of the instrument response function was 140 ps, defining the temporal resolution of our UV microscope.

### FCS Analysis

The fluorescence time trace data were computed with Symphotime 64 (Picoquant) and fitted with Igor Pro 7 (Wavemetrics). The FCS analysis builds on our previous works on nanoantennas in the visible range.^14,28,29,35^ For the rhodium nanoantennas with parallel excitation, the FCS correlations were fitted with a three-species model:^110^
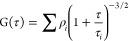
1where ρ_*i*_ and τ_*i*_ are the amplitude and diffusion time of each species, respectively. Here for further implication, we have assumed that the aspect ratio of the axial to transversal dimensions of the detection volume is equal to 1 following our previous works. The rationale behind this three-species model is that the first fast-diffusing species accounts for the molecules inside the nanogap and the second intermediate diffusing species accounts for the molecules present inside the nanorectangle but diffusing away from the nanogap hot spot ,while the third slowly diffusing term is introduced to account for some residual correlation stemming from the background.^48^ For *p*-terphenyl, owing to the fast diffusion time and the high quantum yield of the dye, we find that a two-species model is sufficient to fit the FCS function. For the antennas with perpendicular excitation polarization, we always use only a two-species model, as the nanogap contribution is absent in this case. Typical fit results are detailed in Table S1 in the Supporting Information for the three different target molecules probed here.

Building on our earlier works on plasmonic antennas in the visible,^14,28,29,35^ we use the following notations in our analysis of the antenna’s performance: the average number of molecules present inside the nanogap is N* with a brightness per molecule Q*. The number of molecules diffusing outside the nanogap (but still contributing to the total detected fluorescence and hence to the FCS amplitude) is *N*^0^ with a brightness per molecule *Q*^0^. The total fluorescence intensity is *F*, and *B* is the background intensity recorded on the same nanoantenna in the absence of the target protein. The general FCS formalism in the presence of multiple species^110^ can be inversed to express the number of molecules inside the nanogap N* and their brightness per molecule Q*:
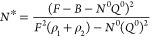
2
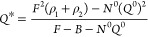
3For the values of the parameters *N*^0^ and *Q*^0^ for the molecules diffusing outside the nanogap, we use the FCS measurements when the excitation polarization is set perpendicular to the dimer axis. We have checked the validity of these values by comparing them with the results obtained with an empty nanorectangle without rhodium nanocubes. The enhancement factor for the fluorescence brightness per molecule is then computed as the ratio between Q* and the reference brightness per molecule value *Q*_ref_ obtained from confocal FCS measurements. In the case of hemoglobin, instead of FCS experiments, we use the known protein concentration and the calibrated confocal volume of 1.8 fL to estimate the number of molecules and their brightness in the diffraction-limited confocal setup.

### Lifetime Analysis

The fluorescence decay histograms were computed and analyzed with Symphotime 64 (Picoquant). We used an iterative reconvolution fit, taking into account the measured instrument response function (IRF). The decay histograms in the nanoantenna were fitted with a three-component exponential model. To ease the comparison between the parallel and perpendicular polarizations, we used the same characteristic lifetimes for both polarizations and computed the intensity-averaged lifetime as the final readout. All the fit parameters are summarized in Table S2.

### Numerical Simulations

The electric field distributions were computed using the wave optics module of COMSOL Multiphysics v5.5, relying on the finite element method. The reflections from the boundaries of the simulation domain were suppressed by using scattering boundary conditions. In our design, we rounded the edges of the rhodium nanocubes with a 5 nm radius of curvature to avoid any spurious effects from sharp edges. The refractive index parameters were taken from predefined libraries of COMSOL Multiphysics. To reproduce the experimental conditions, all of the simulations were performed with the antennas immersed in a water environment on top of the quartz coverslip. To optimize the antenna design and explore a broad range of parameters, we used a 2D model, which was checked to give results comparable to those of a full 3D simulation. We used a tetrahedral user-defined mesh, with mesh size ranging from 0.01 to 10 nm for the 2D model and from 01 to 10 nm for the 3D model. To calculate the excitation intensity enhancement spectra, the rhodium dimer was excited with a plane wave stemming from the quartz substrate with wavelength ranging from 220 to 450 nm. To calculate the radiative rate enhancements, we defined two monitors surrounding the source dipole: one a few wavelengths from the dipole to calculate the radiative power and one only a few nanometers away from the source to calculate the total dissipated power. The antenna influence was determined by comparison with a similar dipolar source near a quartz substrate in water medium in the absence of the rhodium nanocubes. The convergence was checked by generating the error over iteration chart built-in in COMSOL.

## Data Availability

The data that support the findings of this study data are available from the corresponding author upon request.

## Supporting Information Available

Correlative SEM images, comparison with other nanofabrication methods, overview of several antennas, intensity enhancement at 266 and 350 nm, influence of the nanocube tilt, spectral and size dependence, autofluorescence emission spectra, control FCS in the absence of rhodium nanoantenna, control FCS with a single rhodium nanocube, correlation between FCS volume and gap size, brightness enhancement as a function of SEM gap size, nanoantenna enhanced autofluorescence of hemoglobin, fitting parameter results, numerical simulations of decay rate enhancement, photokinetic rates, comparison with aluminum nanogap antennas, and protein information (PDF)
