# Supplementary material for: Ultraviolet Resonant Nanogap Antennas with Rhodium Nanocube Dimers for Enhancing Protein Intrinsic Autofluorescence
Source: ACS Nano. 2023 Nov 6;17(22):22418–29. doi: 10.1021/acsnano.3c05008 (PMC10690780; doi:10.1021/acsnano.3c05008)
Supplement: Supplementary file 1 — nn3c05008_si_001.pdf [file nn3c05008_si_001.pdf]

Supporting Information for

Ultraviolet Resonant Nanogap Antennas with Rhodium Nanocube Dimers for Enhancing Protein Intrinsic Autofluorescence

Prithu Roy,[1] Siyuan Zhu,[2] Jean-Benoît Claude,[1] Jie Liu,[2] Jérôme Wenger[1,*]

[1] Aix Marseille Univ, CNRS, Centrale Marseille, Institut Fresnel, AMUTech, 13013 Marseille, France

[2] Department of Chemistry, Duke University, Durham, NC 27708, USA

** Corresponding author: jerome.wenger@fresnel.fr*

Contents:

S1. Correlative SEM images of rhodium nanogap antennas
S2. Comparison of gap sizes achieved with other nanofabrication methods
S3. Overview of several nanoantennas
S4. Electric field intensity enhancement at 266 nm and at 350 nm
S5. Influence of the nanocube tilt
S6. Spectral and size dependence of the intensity enhancement for different gap sizes
S7. Autofluorescence emission spectra of proteins used in this work
S8. Control FCS in the absence of rhodium nanoantenna
S9. Control FCS with a single rhodium nanocube
S10. Correlation between FCS volume and gap size
S11. Brightness enhancement as a function of SEM gap size
S12. Nano-antenna enhanced autofluorescence of hemoglobin
S13. Fitting parameters results
S14. Numerical simulations of decay rates enhancement
S15. Experimental determination of the photokinetic rates in the rhodium nanoantenna
S16. Comparison with aluminum nanogap antennas
S17. Protein information and sequences

S1. Correlative SEM images of the rhodium nanogap antennas

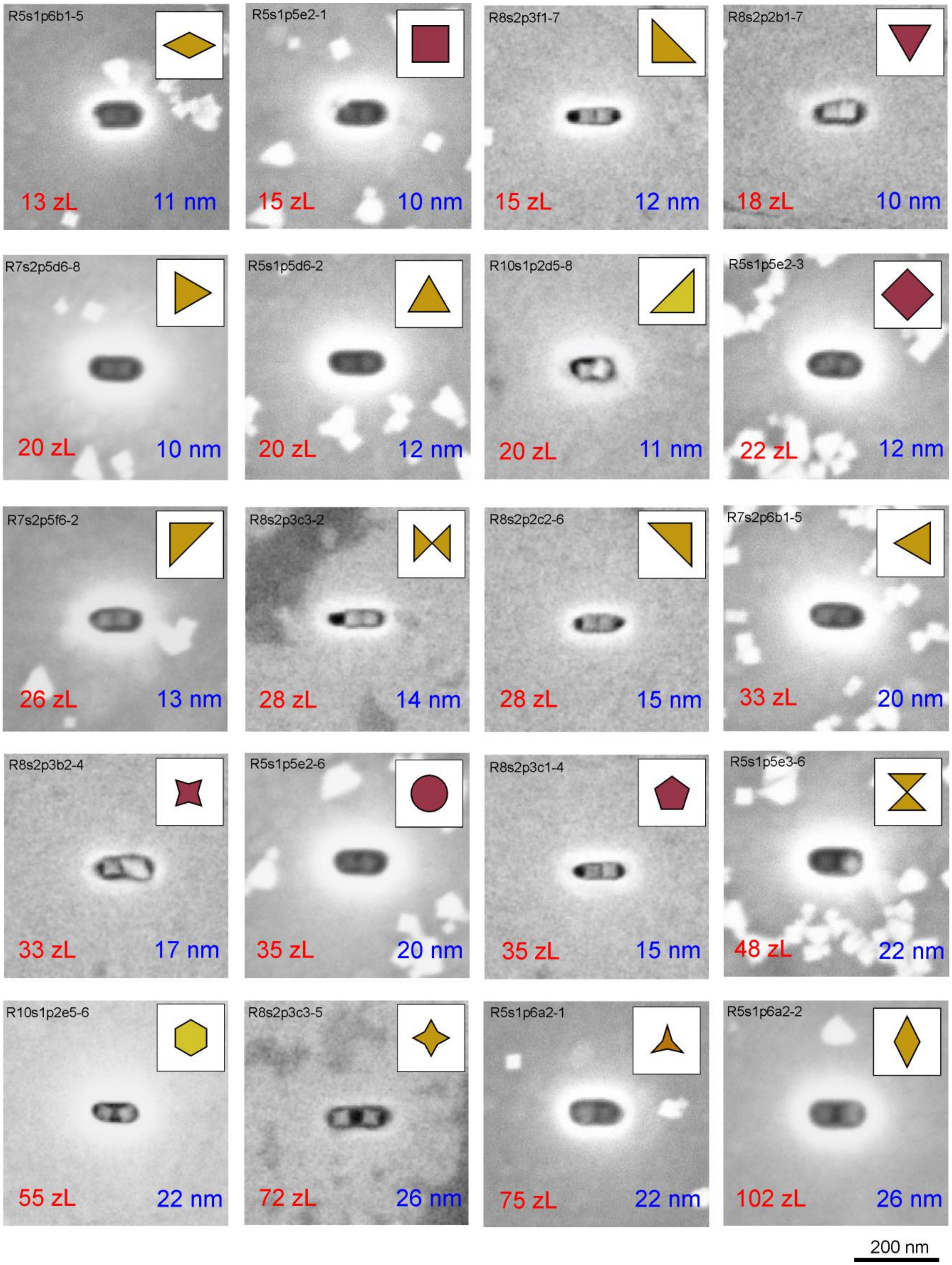

Figure caption appears on next page

Figure S1. Scanning electron microscopy images of the rhodium nanogap antennas used in the experiments corresponding to the data displayed in Fig. 2 & 3. The color symbol in the top right corner of each SEM image corresponds to the symbol used in Fig. 2f & 3d-f so that a direct correlation between FCS results and the actual SEM images can be made. The volume in zeptoliter written in the bottom left of each image is deduced from the FCS measurement of the number of molecules inside the nanogap region and the known molecular concentration. The gap size in the bottom right is obtained from the SEM images as the difference between the total length of the rhodium dimer (measured along the main axis) minus the size of each rhodium nanocube (measured along the direction perpendicular to the main axis). For a correlation between the gap sizes deduced from FCS and SEM, please refer to Fig. S10. The nanoantennas are ranked from top left to bottom right as a function of the FCS volume. The alphanumeric code in the top left of each image is our internal reference of each antenna. As the nanocubes forming the antenna are positioned inside the rectangular aperture and are thus below the aluminum surface, they appear dimmer than the other rhodium nanocubes and triangular nanoparticles dispersed on top of the aluminum surface.

S2. Comparison of gap sizes achieved with other nanofabrication methods

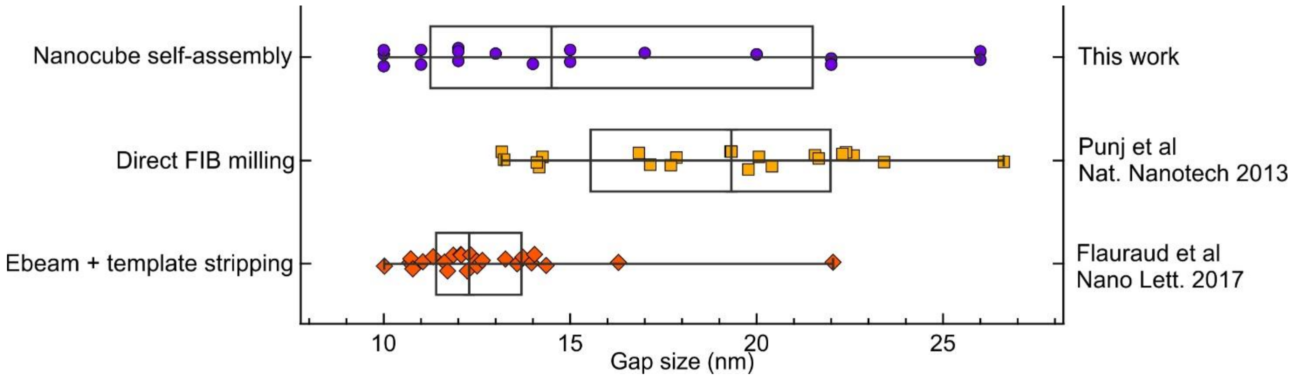

Figure S2. Comparison of the estimated gap sizes achieved using different fabrication methods. The markers represent individual nanoantennas while the boxes and whiskers display the median, 25th and 75th percentile and the min/max values. For focused ion beam (FIB) and electron beam lithography (Ebeam) we refer to past works from our group on gold nanogap antennas. This comparison demonstrates that the gap sizes achieved using self-assembly into nanorectangles are quite comparable with other nanofabrication methods. Moreover, let us stress that the main focus of this work is to demonstrate the realization of ultraviolet nanogap antennas and assess their performance for the detection of label-free proteins. We are not aiming at developing a new nanofabrication method.

S3. Overview of several nanoantennas

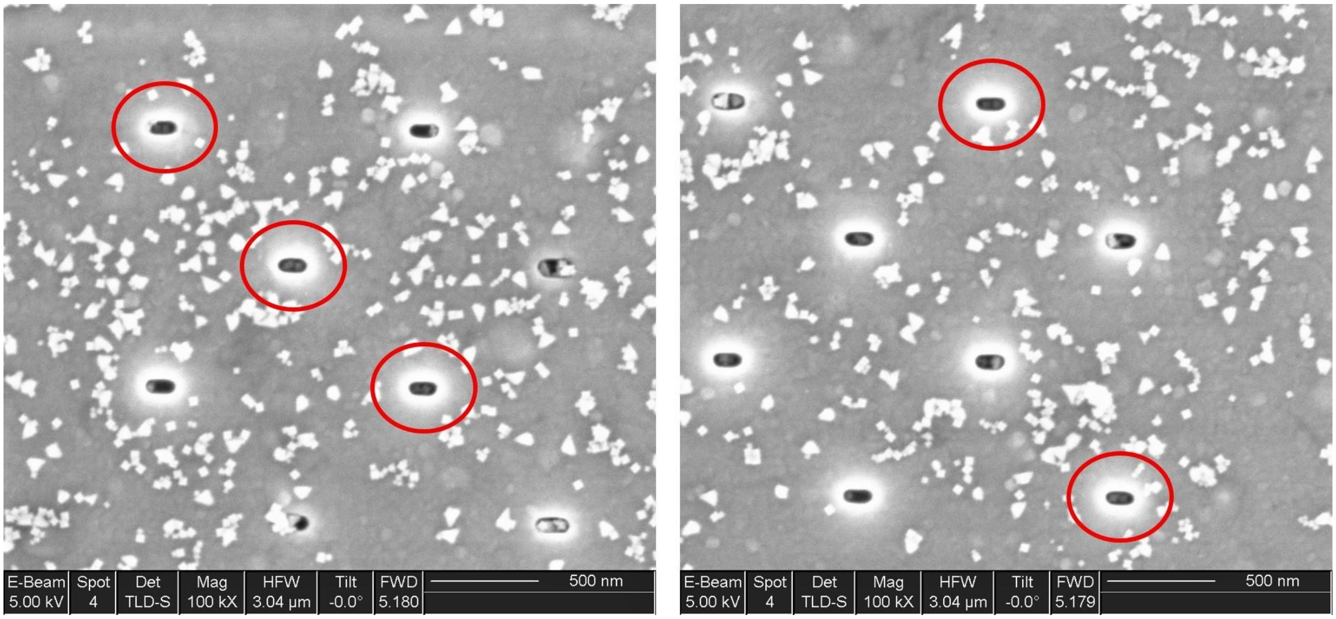

Figure S3. SEM images overview of two zones featuring 8 rectangular nanoapertures milled into the aluminum film. The apertures circled in red have two rhodium nanocubes assembled into a nanogap antenna, which are selected for the UV fluorescence experiments. The other apertures are discarded. By doing correlative measurements between the electron microscope and the UV microscope, we can select the self-assembled dimer nanogap antennas. After rinsing, the nanoantennas can be reused several times.

S4. Electric field intensity maps at 266 nm and at 350 nm

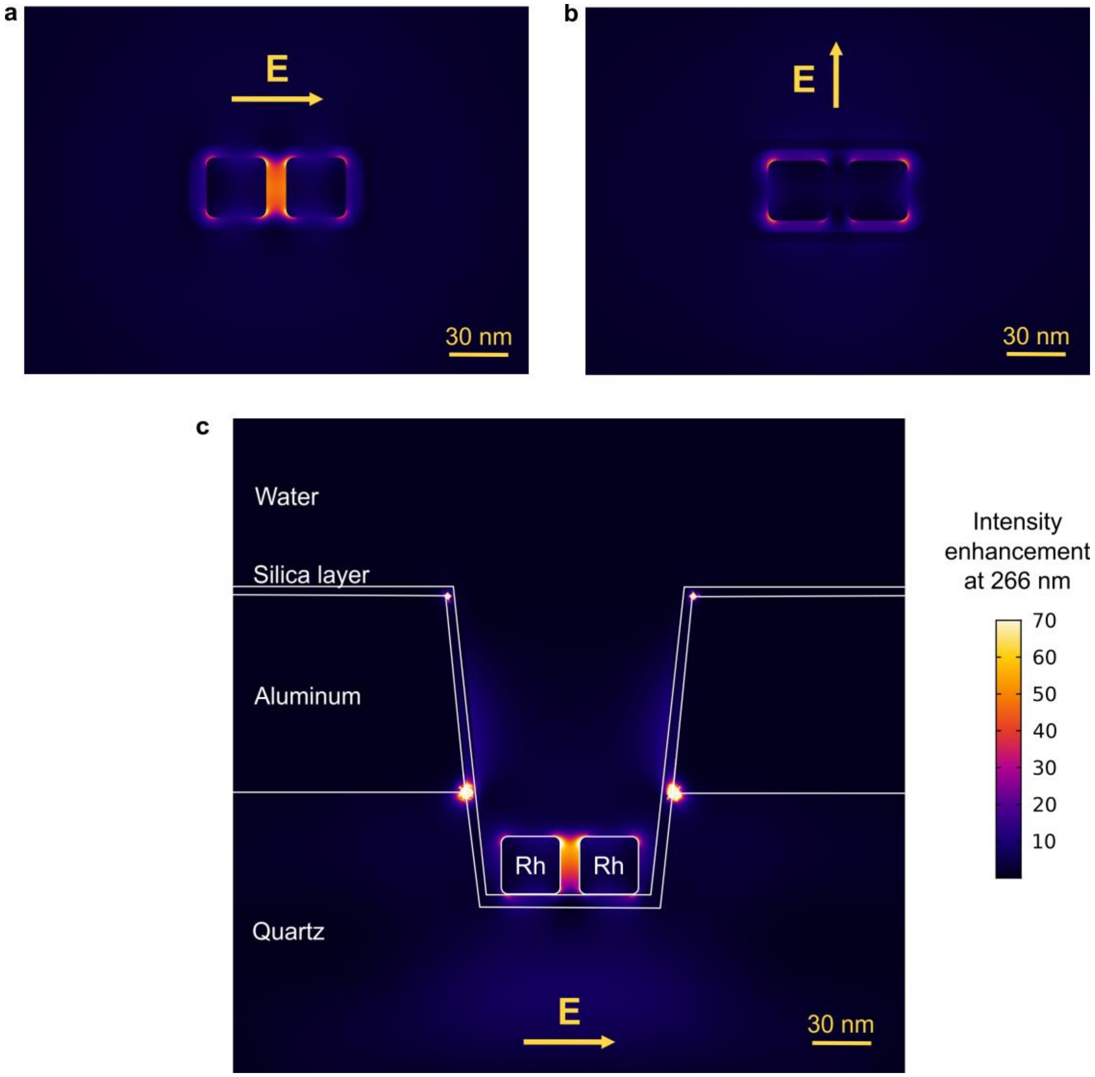

Figure S4. 3D numerical simulations of the electric field intensity enhancement at 266 nm for a UV nanogap antenna made of two 30 nm rhodium cubes separated by a 10 nm gap inserted in a rectangular aperture milled into an aluminum film. The arrows indicate the orientation of the incident electric field. The maps a,b are taken 5 nm below the top surface of the nanocubes. All the maps share the same colorscale.

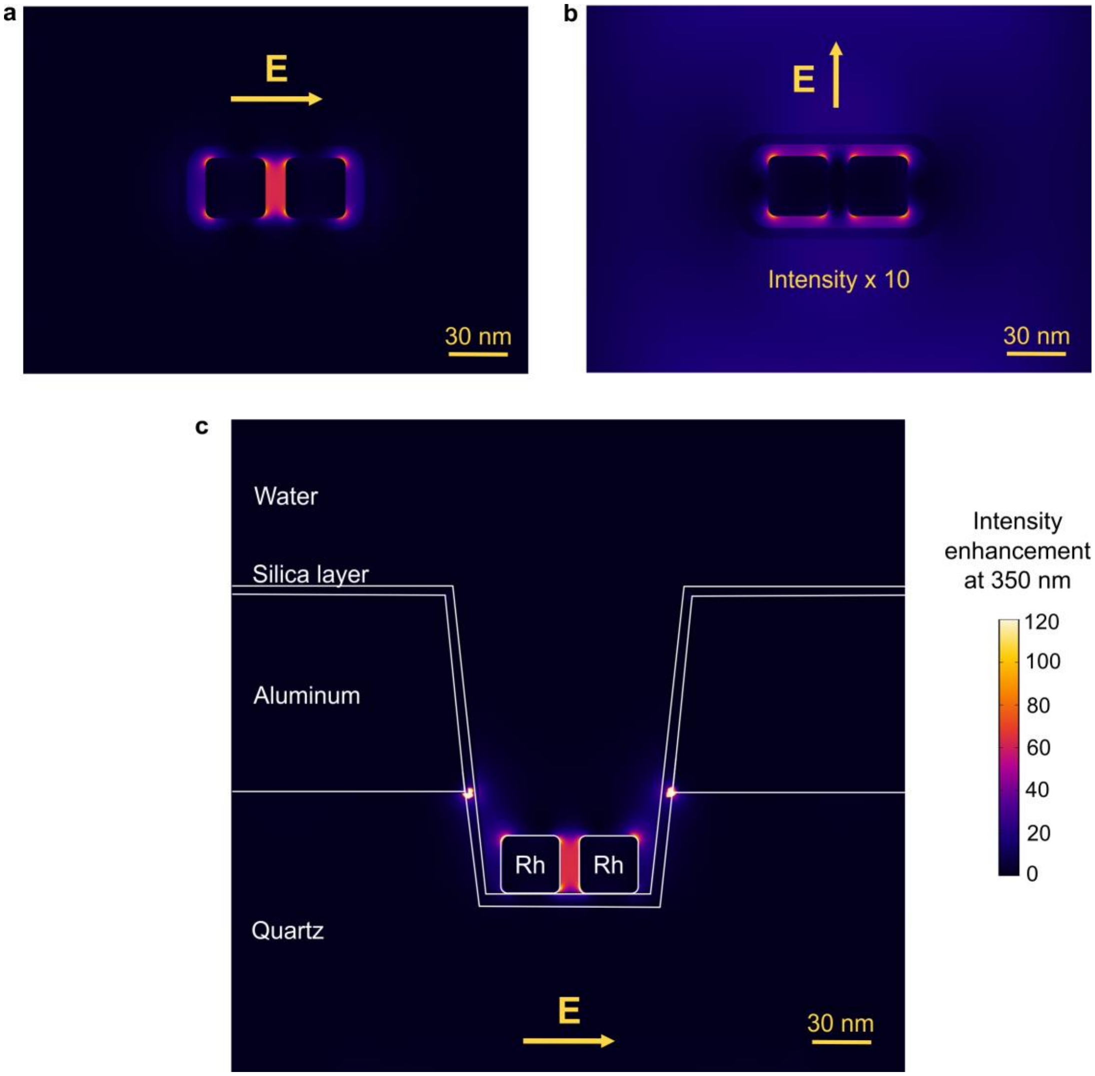

Figure S5. 3D numerical simulations of the electric field intensity enhancement at 350 nm for a UV nanogap antenna made of two 30 nm rhodium cubes separated by a 10 nm gap inserted in a rectangular aperture milled into an aluminum film. The arrows indicate the orientation of the incident electric field. The maps a,b are taken 5 nm below the top surface of the nanocubes. So that all the maps share the same colorscale, we have multiplied the intensity for the perpendicular orientation (b) by a constant value of 10x.

S5. Influence of the nanocube tilt

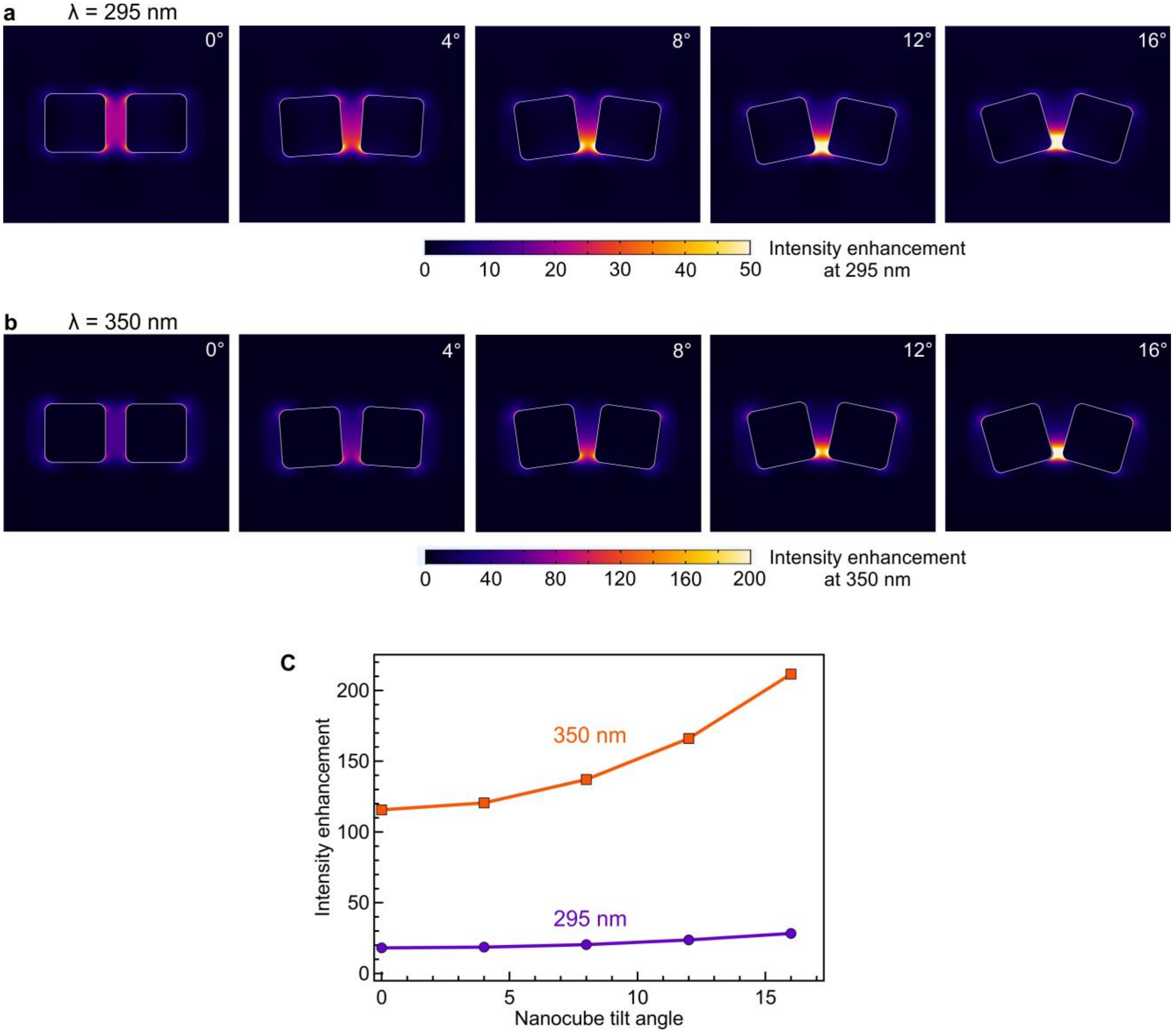

Figure S6. Influence of non-perfectly aligned nanocubes on the nanoantenna optical performance. (a) Spatial distributions of the intensity enhancement at 295 nm for different tilt angle of the nanocubes. The angle indicated in the top right corner of each image correspond to the rotation angle of each nanocube in the horizontal XY plane. Each nanocube is rotated by this angle with opposite rotation directions. The nanocube size is 30 nm and their center-to-center distance is kept constant at 40 nm, which corresponds to a 10 nm gap for perfectly aligned nanocubes. (b) Same as (a) for 350 nm wavelength. (c) Spatially-averaged intensity enhancement along a 30 nm vertical line in the middle of the nanogap between the nanocubes.

S6. Spectral and size dependence of the intensity enhancement for different gap sizes

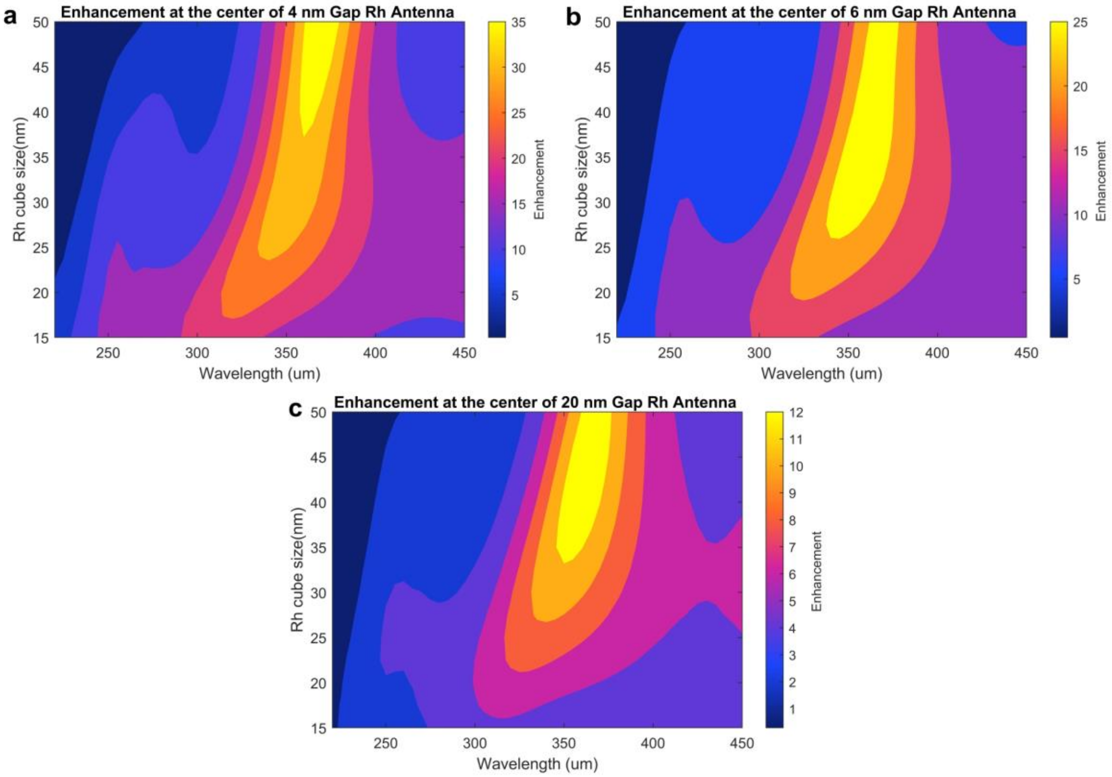

Figure S7. Numerical simulations of the spectral dependence of the intensity enhancement in the center of the nanogap antenna as a function of the rhodium nanocube size and gap size. For (a-c) the gap sizes are respectively set to 4, 6 and 20 nm. To speed up the numerical calculations and provide design guidelines, the simulations consider a pair of rhodium nanocubes on a quartz coverslip immersed in water without aluminum layer, so the maximum enhancement values are lower by typically a factor ~4x as compared to the full 3D simulations including the aluminum nanorectangle (Fig. S4 and S5). Zero-mode waveguides and nanorectangle apertures are known to locally enhance the electromagnetic field intensity.[1]

S7. Autofluorescence emission spectra of proteins used in this work

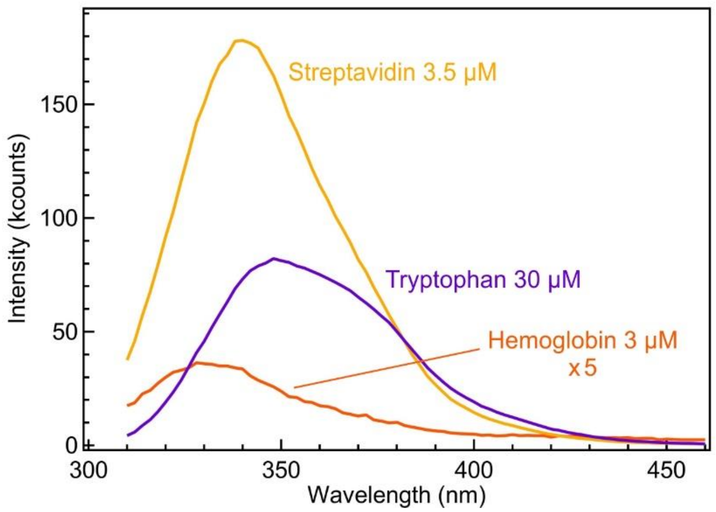

Figure S8. Autofluorescence emission spectra of streptavidin and hemoglobin solutions recorded along with a solution of tryptophan diluted in water to serve as a quantum yield reference. The spectra were recorded on a Tecan Spark 10M spectrofluorometer with 260 nm excitation and identical fluorescence detection conditions. The intensity for the hemoglobin spectrum has been multiplied by 5 times to ease viewing on the same graph. To estimate the average quantum yield of the protein autofluorescence, we compute the ratios of the fluorescence intensities integrated over the 310-410 nm spectral region, normalized by the absorbance of the same solutions measured at 260 nm, and we use the calibrated 12% quantum yield of tryptophan in water 100 mM phosphate buffer solution.[2]

S8. Control FCS in the absence of rhodium nanoantenna

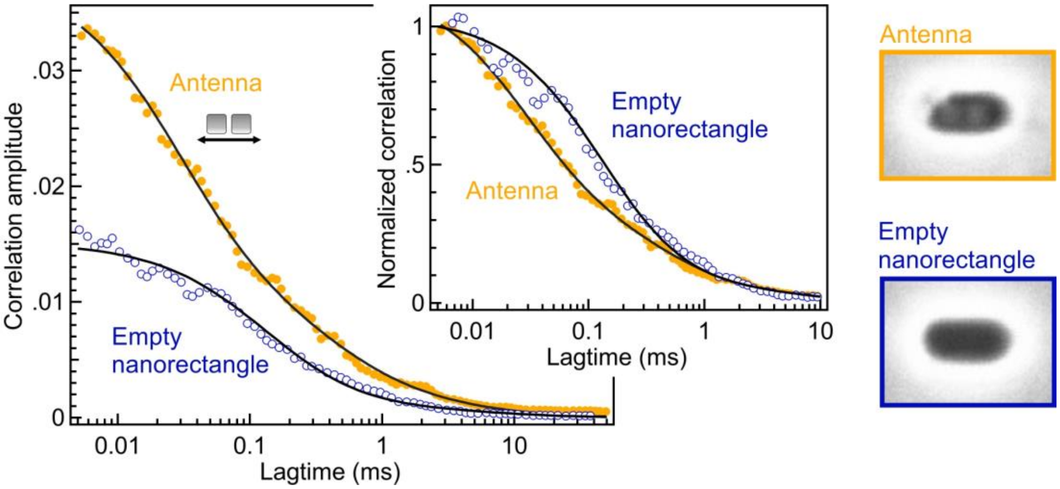

Figure S9. Comparison between the FCS correlation functions obtained for a dimer nanoantenna and a nanorectangular aperture without rhodium nanocubes. The conditions are identical to the ones used for Fig. 2 with streptavidin proteins. Dots are experimental data, lines are numerical fits. The insert graph shows the amplitude-normalized FCS functions to highlight the shorter diffusion time in the case of the nanogap antenna. For the experiments on the empty nanorectangle, we recorded an average total intensity F of 8030 counts/s and a background B of 2200 counts/s. From the FCS fit amplitude ρ_1 of 0.014, we deduce a number of molecules in the nanorectangle of $N = \left(1 - \frac{B}{F}\right)^2 \frac{1}{\rho_1}$ of 36.8 molecules with a brightness $(F - B)/N$ of 158 counts/s, which is enhanced by 7.2× above the reference 22 counts/s found for streptavidin on our confocal setup.

S9. Control FCS with a single rhodium nanocube

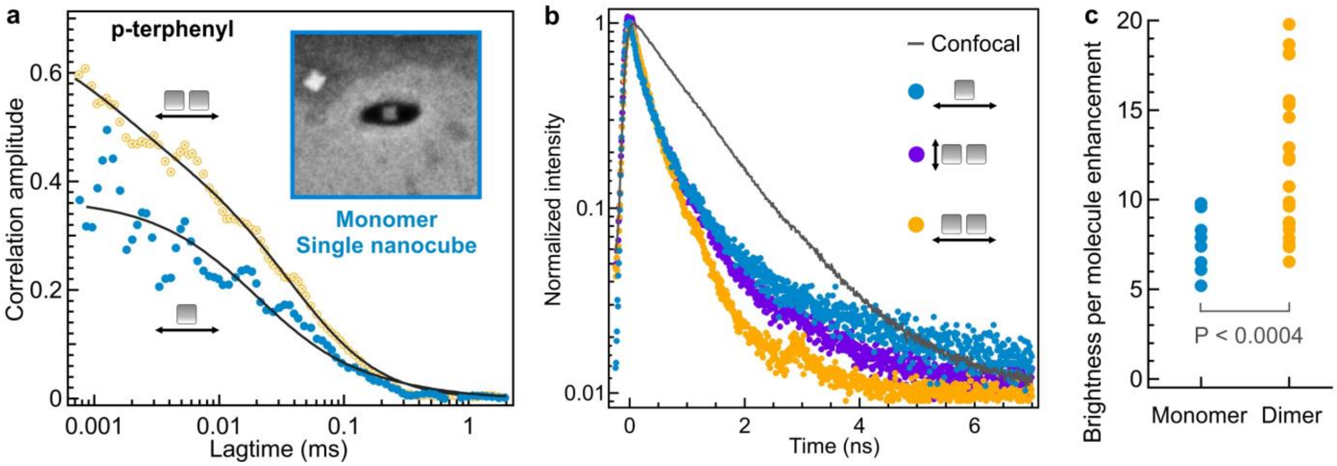

Figure S10. Control FCS experiment with a single rhodium nanocube. The conditions are identical to the ones used for Fig. 3 for p-terphenyl dissolved in a glycerol:ethanol mixture (60:40 volume ratio) to increase the viscosity, slow down the diffusion time and facilitate the FCS measurement. The concentration is 10 µM, the 266 nm excitation power is 40 µW. (a) FCS correlation functions. The average total intensity F is 4570 counts/s and the background B is 1700 counts/s. From the FCS fit amplitude ρ_1 of 0.34, we deduce a number of molecules of $N = \left(1 - \frac{B}{F}\right)^2 \frac{1}{\rho_1}$ of 1.2 molecules with a brightness $(F - B)/N$ of 2400 counts/s, which is enhanced by 5.2× above the reference 475 counts/s found for p-terphenyl on our confocal setup. (b) Comparison of the normalized time-resolved decay traces, superposing to the data in Fig. 3 the result for the single rhodium nanocube. A slightly longer fluorescence lifetime is observed in the case of the single rhodium cube, which may be related to a reduced quenching rate with a single nanocube instead of two. (c) Comparison of the fluorescence enhancement factors for the brightness per emitter for the single nanocube (monomer) and the dimer of nanocubes, both with parallel excitation. A statistical T-test has been performed to compare the distributions, the resulting P value is written in the graph. The null hypothesis is clearly rejected.

S10. Correlation between FCS volume and gap size

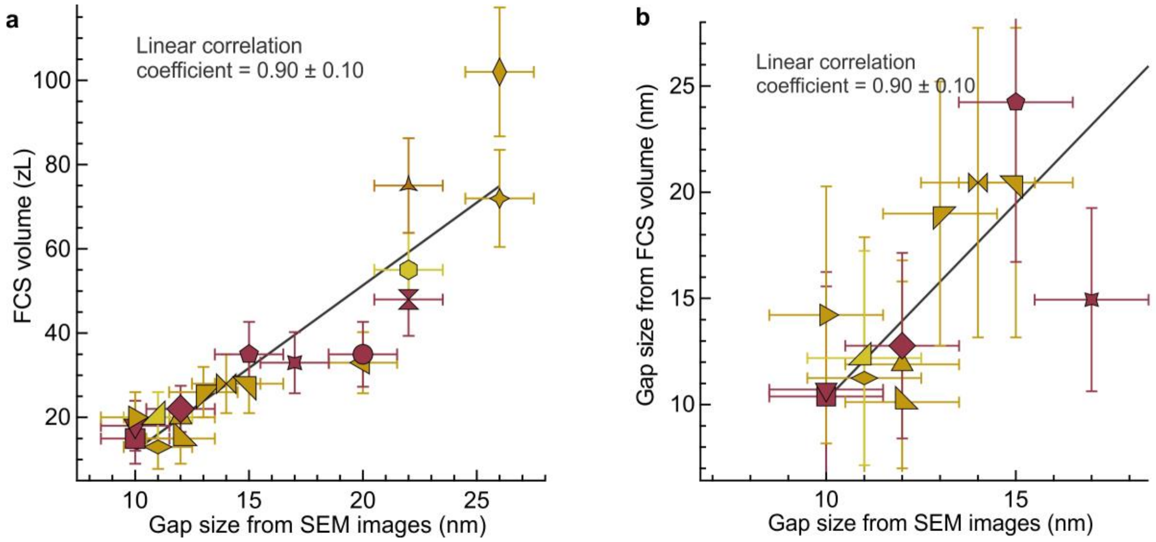

Figure S11. Correlation between the gap size obtained from the SEM images with the volume measured with FCS (a) or the gap size deduced from the FCS volume (b) for a selection of the antennas with the smallest gaps. The SEM gap size is the same as the one indicated on Fig. S1. It is obtained from the SEM images as the difference between the total length of the rhodium dimer (measured along the main axis) minus the size of each rhodium nanocube (measured along the direction perpendicular to the main axis). The FCS volume (a) is derived from the FCS measurement of the number of molecules inside the nanogap region and the known molecular concentration. To estimate the gap size from the FCS volume (vertical axis in b), we divide the FCS volume by the lateral area of the rhodium nanocube (square of the average nanocube size measured from the FCS images along the perpendicular direction plus a constant 6 nm to account for the expansion of the detection volume beyond the geometrical limits of the nanocube). Linear fits and Pearson correlation coefficient are indicated on each graph. These results demonstrate the correlation between the FCS results and the SEM gap sizes.

S11. Brightness per molecule enhancement as a function of gap size from SEM images

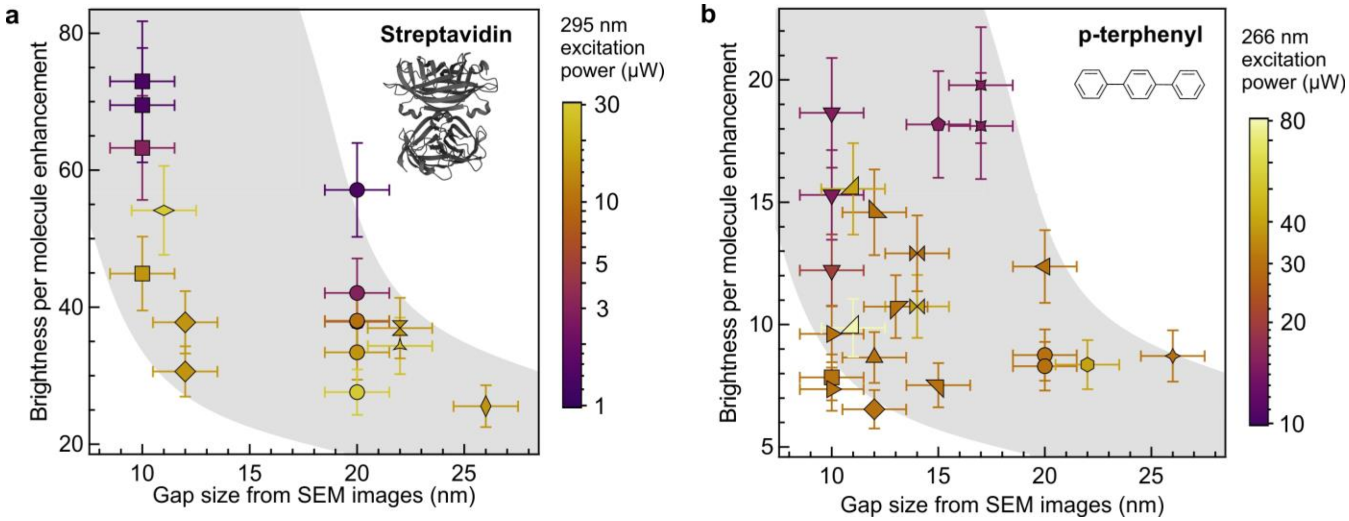

Figure S12. Scatter plot of the fluorescence brightness enhancement for streptavidin (a) and p-terphenyl (b) as a function of the gap size deduced from the SEM images in Fig. S1. The enhancement values are the same as in Fig. 2f and 3d, yet the x-axis variable is now the gap size deduced from the SEM images instead of the number of molecules in the nanogap measured with FCS. The different markers indicate the different nanoantennas (same code as in Fig. S1), and the color indicates the excitation power used. The shaded areas are guides to the eyes.

S12. Nano-antenna enhanced autofluorescence of hemoglobin

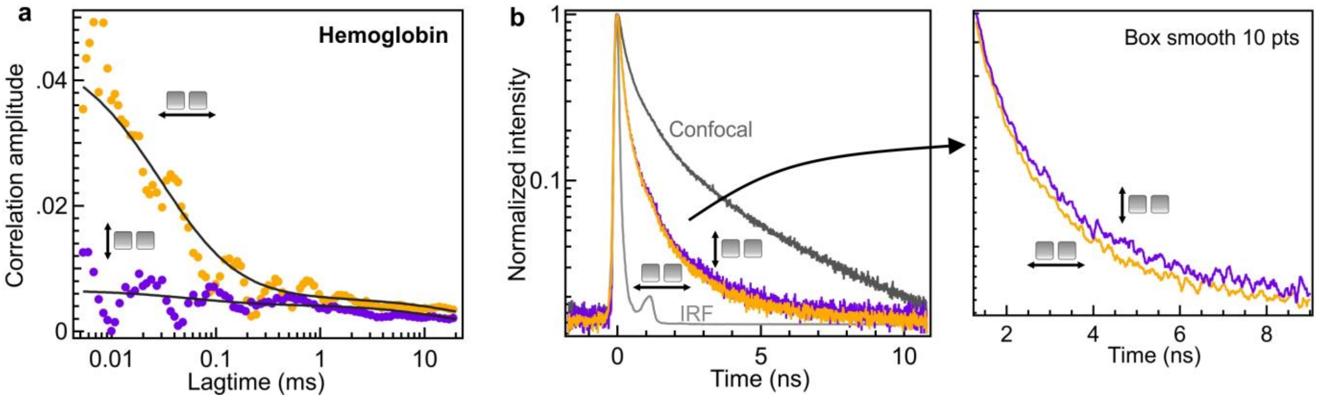

Figure S13. FCS and TCSPC data for label-free hemoglobin in a nanogap antenna. The antenna used for this data has the reference number R5s1p5e2-6 for which a 20 nm gap was deduced from the SEM image (Fig. S1). (a) FCS correlation functions, dots are experimental data, lines are numerical fits. The antenna is covered with a 50 µM solution of diffusing label-free hemoglobin proteins. The 295 nm excitation power used here is 15 µW. (b) Normalized time-resolved decay traces corresponding to the data in (a) and to the confocal reference (dark gray). IRF stands for the instrument response function. The insert graph on the right is a close-up view of the decays for excitation polarization set parallel and perpendicular to the dimer's main axis. The data have been smoothed with 10 points box averaging.

S13. Fitting parameters results

Table S1. Fitting parameters for the FCS data.

Protein	STREPTAVIDIN		P-TERPHENYL		HEMOGLOBIN	
Concentration	50 µM		10 µM		50 µM	
Power (µW)	15		40		15	
Exc. Polarization	Parallel	Perpendicular	Parallel	Perpendicular	Parallel	Perpendicular
F (counts/s)	5740	4860	3370	2100	4070	2970
B (counts/s)	2100	2100	1700	1450	2200	2200
ρ_1	0.0276	--	--	--	0.028	--
ρ_2	0.0093	0.0145	0.635	0.119	0.012	0.0024
ρ_3	0.0014	--	0.034	0.027	0.0053	0.0042
τ_1 (ms)	0.042	--	--	--	0.04	--
τ_2 (ms)	0.70	0.73	0.005	0.0066	0.07	0.08
τ_3 (ms)	8	--	1.4	4.6	49	30
G(0) = $\rho_1 + \rho_2$	0.0369	0.014	0.635	0.119	0.040	0.0024
N_0	22.2	--	0.8	--	28	--
Q_0 (counts/s)	124	--	807	--	27.5	--
N*	0.90	22.2	0.16	0.8	1.9	28
Q* (counts/s)	987	124	6530	807	583	27.5
Q_{ref} (counts/s)	22	22	475	475	3.5	3.5
Brightness enhancement	44.9	5.6	13.75	1.7	166	7.9

Table S2. Fitting parameters for the TCPSC data. For the average lifetime of p-terphenyl in parallel case, we took only the first and second component into consideration to compute the intensity-averaged lifetime.

Protein	STREPTAVIDIN		P-TERPHENYL		HEMOGLOBIN	
Exc. Polarization	Parallel	Perpendicular	Parallel	Perpendicular	Parallel	Perpendicular
τ_1 (ns)	0.025		0.025		0.025	
τ_2 (ns)	0.166		0.140		0.243	
τ_3 (ns)	0.860		0.450		1.740	
I_1 (%)	19.74	23.91	7.48	30.85	11.09	18.52
I_2 (%)	32.61	23.91	25.99	--	60.27	52.15
I_3 (%)	47.64	52.18	66.53	69.15	28.64	29.33
$\tau_{average\ intensity}$ (ns)	0.47	0.49	0.114	0.32	0.65	0.64

S14. Decay rates enhancement with rhodium nanogap antennas

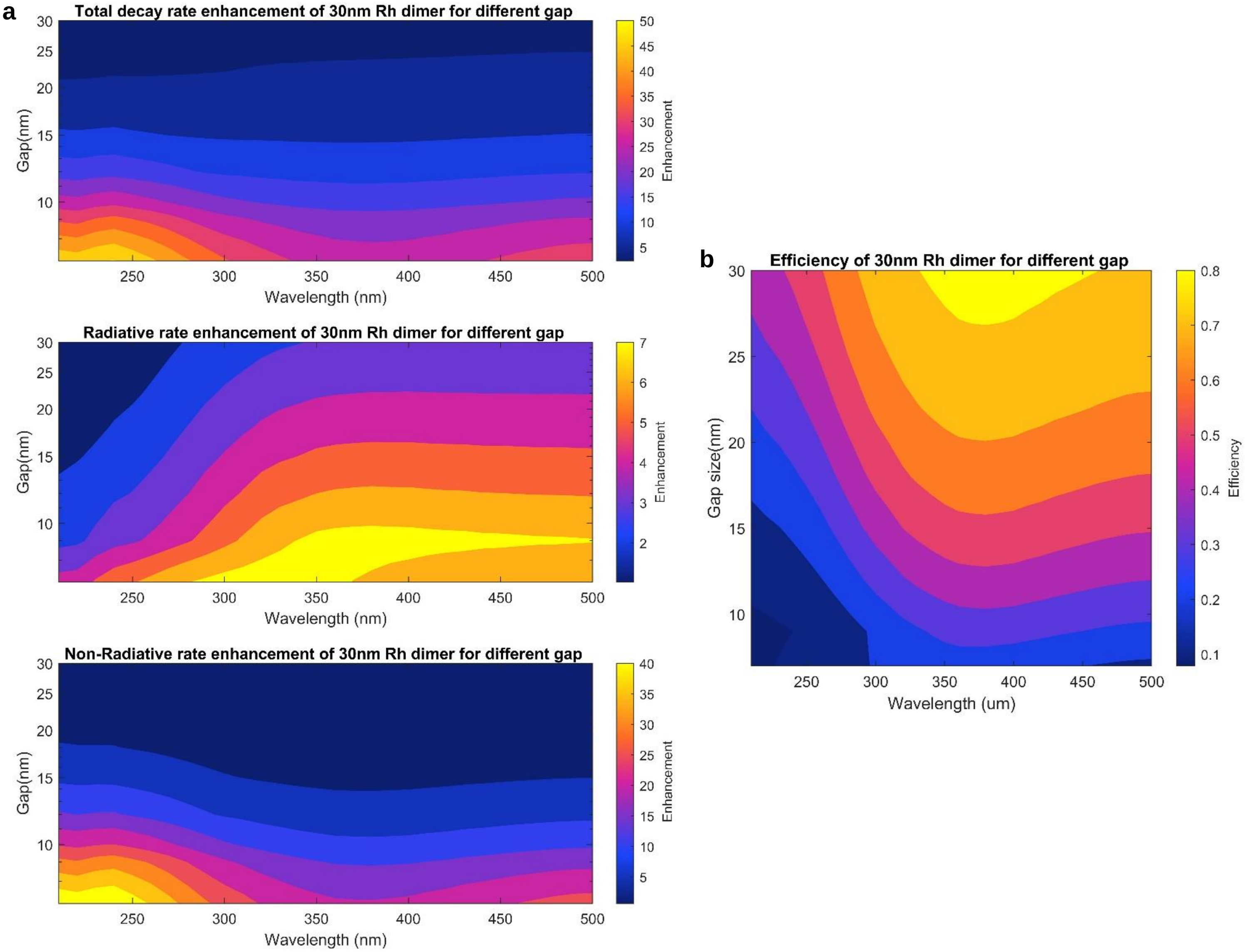

Figure S14. (a) Numerical simulations of the total, radiative and nonradiative decay rate constant enhancement for a point dipole located in the center of a rhodium dimer nanogap antenna oriented along the antenna's main axis as a function of the emission wavelength and the gap size. The cube size is constant at 30 nm. All rates are normalized to the dipole's radiative rate in free space. (b) Simulations of the antenna radiative efficiency (ratio of radiative rate to total decay rate) as a function of the emission wavelength and the gap size for a perfect point dipole emitter with parallel orientation located in the center of the nanogap. The rhodium cube size is constant at 30 nm.

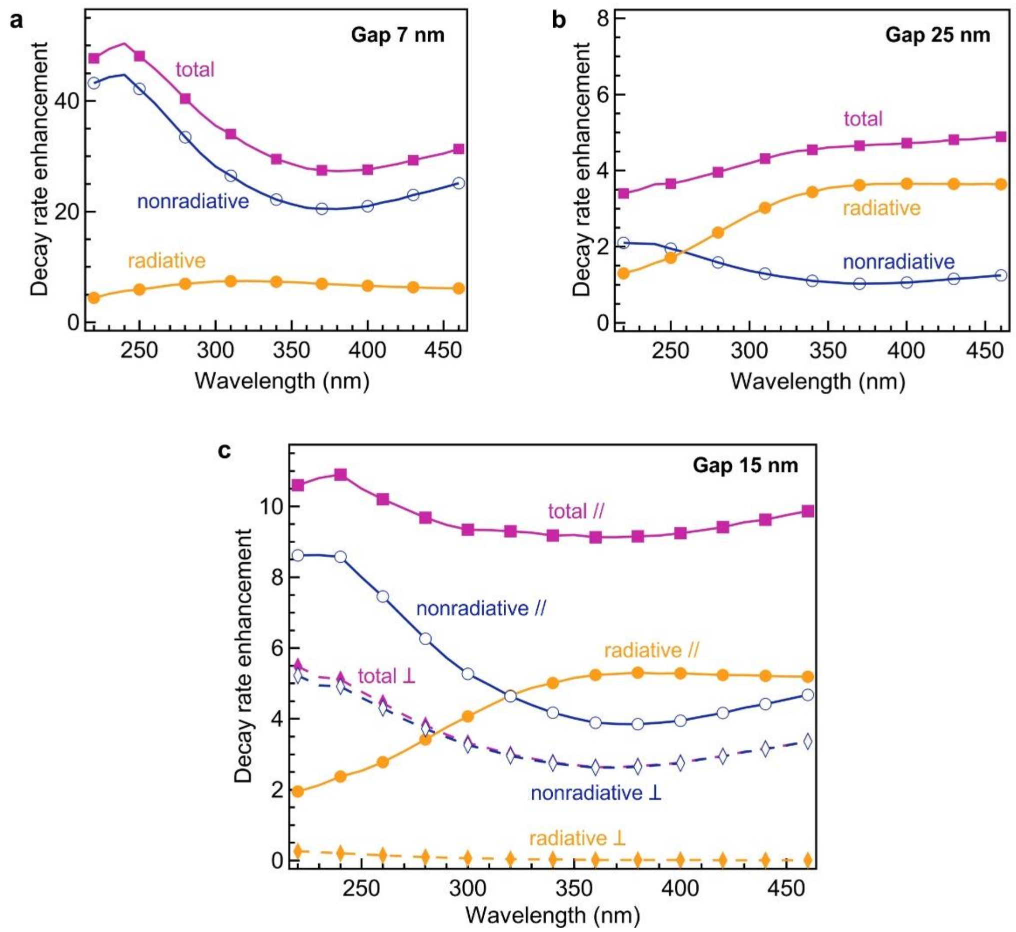

Figure S15. (a-c) Numerical simulations of the enhancement of the decay rate constants as a function of the emission wavelength for a perfect point dipole emitter with parallel orientation located in the center of the nanogap. In (c), the enhancement factors for a dipole with perpendicular orientation are displayed with dashed lines and diamond markers. The rhodium cube size is constant at 30 nm. The gap size is 7 nm in (a), 25 nm in (b) and 15 nm in (c). All rates are normalized respective to the dipole radiative rate in free space.

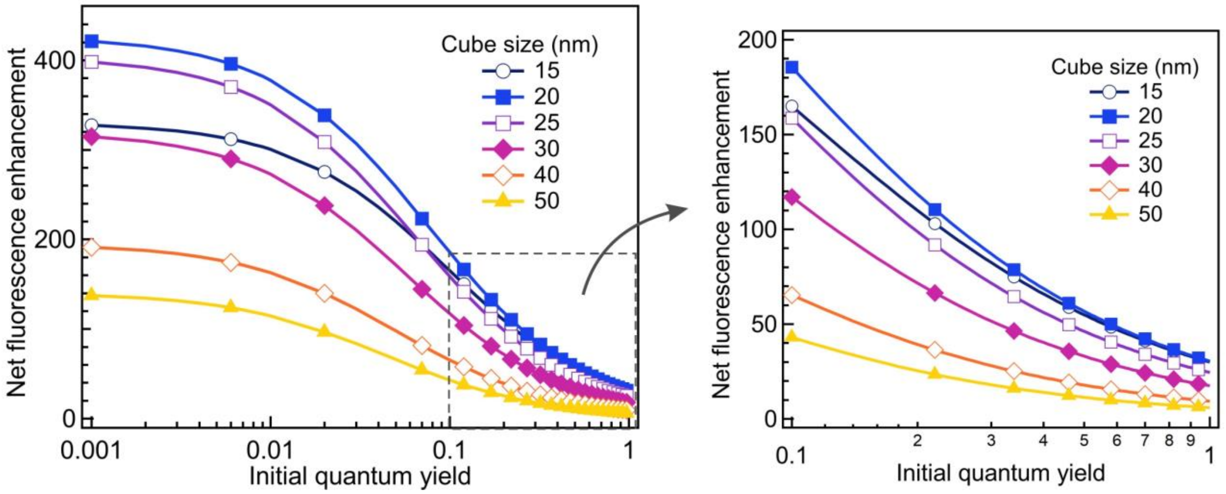

Figure S16. Simulations of the maximum fluorescence brightness enhancement as a function of the initial quantum yield for a point dipole located in the center of the nanogap with an orientation parallel to the dimer's main axis. The gap size is kept constant at 10 nm. The excitation wavelength is 295 nm and the emission is 350 nm. The right panel is a close-up view of the zone with quantum yields between 0.1 and 1.

S15. Experimental determination of the photokinetic rates in the rhodium nanoantenna

To determine experimentally the influence of the rhodium nanoantenna on the photokinetic rates, we use the following approach and notations: for the confocal reference, the total decay rate constant $\Gamma_{tot}^{0} = \Gamma_{rad}^{0} + \Gamma_{nr}^{0}$ is the sum of the radiative Γ_{rad}^{0} and nonradiative Γ_{nr}^{0} decay rate constants. It also amounts to the inverse of the fluorescence lifetime $1 / \tau_0$. The quantum yield is $\phi_0 = \Gamma_{rad}^{0}/\Gamma_{tot}^{0}$. The different values are summarized in Table S3 for the various molecules used here.

In presence of the nanoantenna, the lifetime is shortened, and becomes $1 / \tau^* = \Gamma_{rad}^{*} + \Gamma_{nr}^{0} + \Gamma_{loss}^{*}$. Here we consider that the radiative decay rate constant Γ_{r}^{*} is enhanced (Purcell effect), that the internal nonradiative decay rate constant Γ_{nr}^{0} is unaffected by the photonic environment and that an additional nonradiative decay channel Γ_{loss}^{*} is introduced to account for the extra losses into the free electron cloud in the metallic antenna.[3,4]

The fluorescence brightness enhancement η_F corresponds to the product of the gains in excitation intensity η_{exc}, quantum yield η_{ϕ}, and collection efficiency η_{coll}.[5] The quantum yield gain can be further written as the ratio between the gains in the radiative rate $\eta_{\Gamma rad} = \Gamma_{rad}^{*}/\Gamma_{rad}^{0}$ and the total decay rate $\eta_{\Gamma tot}$= $\Gamma_{tot}^{*}/\Gamma_{tot}^{0}$, so the fluorescence enhancement becomes $\eta_F = \eta_{exc}\, \eta_{coll}\, \eta_{\Gamma rad} \,/\, \eta_{\Gamma tot}$ (here $\eta_{\Gamma tot}$ is also equivalent to the reduction in the fluorescence lifetime). Using these notations, the fluorescence enhancement can be rewritten to clearly show the dependence with the initial quantum yield ϕ_0:[6–8]

$$\eta_F = \eta_{exc}\, \eta_{coll}\, \eta_{\Gamma rad} \frac{1}{1 - \phi_0 + \phi_0\, (\Gamma_{rad}^{*} + \Gamma_{loss}^{*})/\Gamma_{rad}^{0}}$$

With the 0.8 numerical aperture of our microscope objective, the laser beam can still be considered to be moderately focused (this numerical aperture corresponds to a maximum angle of 34° in quartz). In this case, the reciprocity theorem states that the gain in excitation intensity amounts to the products of the gains in collection efficiency times the gain in radiative rate: $\eta_{exc} = \eta_{coll}\, \eta_{\Gamma rad}$.[9] We also use recent numerical simulations of the collection efficiency gain to estimate its value to $\eta_{coll} = 1.44$ for all the different molecules here.[10]

From the measurements of the brightness enhancement η_F together with the fluorescence lifetimes in confocal and in the nanoantenna and the knowledge of the quantum yield ϕ_0 in homogeneous solution, we can compute back all the different rate constants, including the losses to the metal Γ_{loss}^{*}. The main results are summarized in Table S3. Interestingly, despite more than two orders of magnitude difference in the initial quantum yields of p-terphenyl, streptavidin and hemoglobin, we find consistent results in the excitation gain, radiative gain and loss decay rate, indicating a common electromagnetic origin for these effects. The orientation-averaged and position-averaged excitation gain $\eta_{exc} = 15.5 \pm 3.8$ appears in correct agreement with the numerical simulations Figs. 1c-f, as well as the radiative gain $\eta_{\Gamma rad} = 10.8 \pm 2.6$. The loss rate constant $\Gamma_{loss}^{*} = 1.25 \pm 0.3$ ns^{-1} appears to be also a preserved feature among our different experiments.

Table S3. Photokinetic rate parameters. The decay rate constants are expressed in ns^{-1}, the lifetimes are in ns.

	ϕ_0	τ_0	Γ_{tot}^{0}	Γ_{rad}^{0}	Γ_{nr}^{0}	η_F	τ^*	η_{exc}	$\eta_{\Gamma rad}$	Γ_{loss}^{*}	ϕ^*	η_ϕ
P-ter phenyl	0.93	1.00	1.00	0.93	0.07	16.1	0.11	12.4	8.6	1.5	0.84	0.9
Strep tavidin	0.035	1.50	0.67	0.02	0.64	65	0.47	14.4	10.0	1.3	0.11	3.1
Hemo globin	0.005	2.10	0.48	0.00	0.47	120	0.65	19.7	13.6	1.0	0.021	4.2

S16. Comparison of the enhancement factors with aluminum nanogap antennas

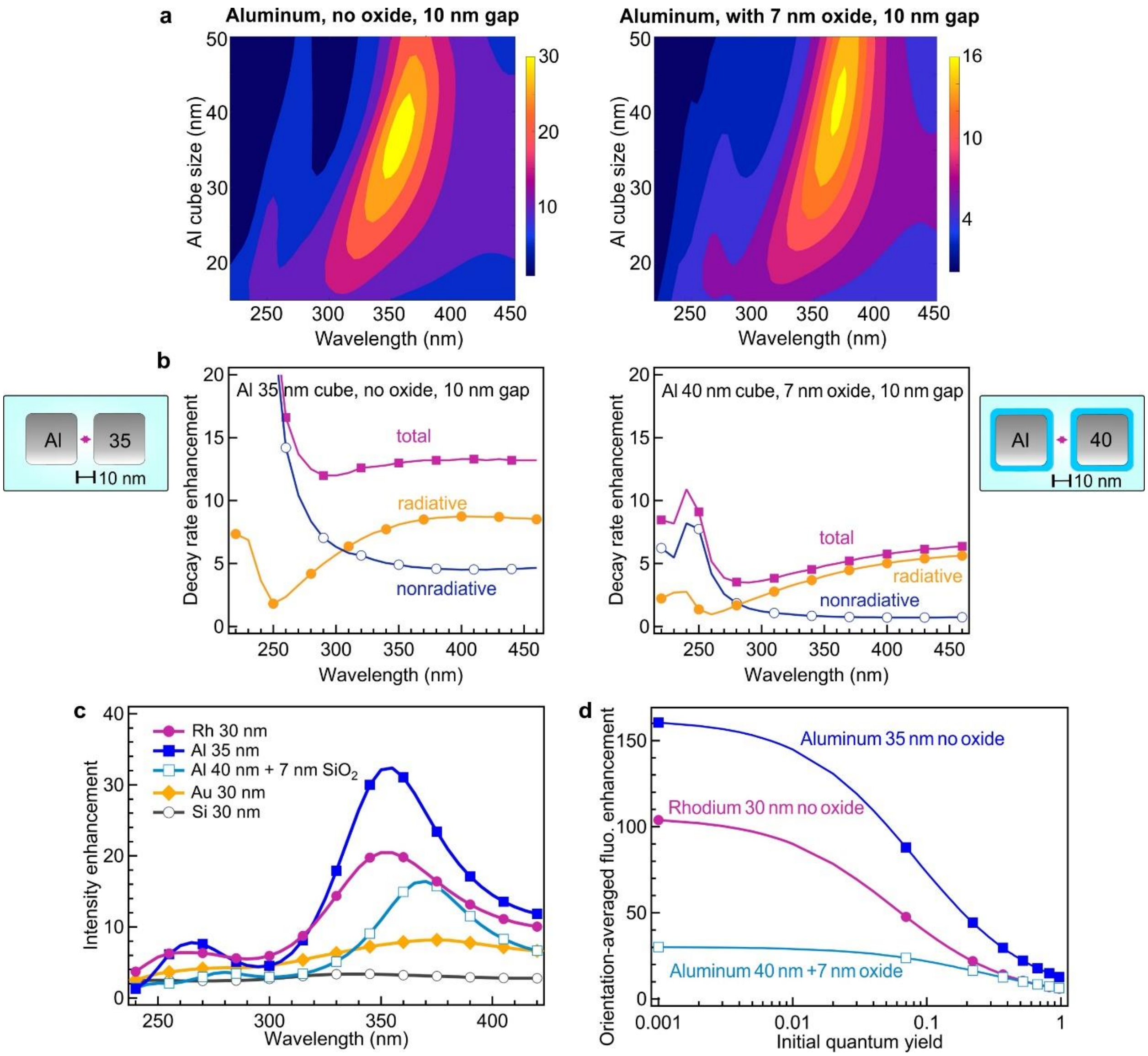

Figure S17. Comparison of rhodium and aluminum nanocube antennas in water. (a) Spectral dependence of the intensity enhancement in the center of the nanogap as a function of the nanocube size. The gap is constant at 10 nm. The left image is for pure aluminum without any oxide layer. On the right image, we have added a 7 nm thick conformal silica layer surrounding the nanocube to simulate the influence of an extra corrosion protection layer. Corrosion of aluminum in water environment is a major issue, especially under UV illumination.[11–14] Based on the results in (a), we select a nanocube size of 35 nm for pure aluminum, and a 40 nm size in the presence of silica. (b) Decay rate enhancement for a perfect dipole emitter with parallel orientation located in the center of the 10 nm nanogap. All rates are normalized respective to the dipole radiative rate in free space. (c) Comparison of the intensity enhancement in the center of the 10 nm gap between nanocubes made of different materials. The nanocube sizes and the materials are indicated in the figure legend. We have considered amorphous silicon. (d) Comparison of the net fluorescence brightness enhancement for three different materials as a function of the emitter's initial quantum yield in free space. The gap size is kept constant at 10 nm. The excitation wavelength is 295 nm for rhodium and 266 nm for aluminum since this wavelength gives a slightly better overall enhancement. The emission wavelength is 350 nm and is averaged over the three orientation directions.

S17. Protein information and sequences

Table S4. Information about the proteins used in this work.

Name	**Streptavidin**	**Hemoglobin**	
Organism	Streptomyces avidinii	Homo Sapiens (Human)	
UniPROT reference	P22629	α subunit: P69905	β subunit: P68871
RCSB PDB structure ref	2RTR	6BB5	
Sigma Aldrich product number	S4762	H7379	
Form	Homotetramer	Heterodimer of α and β subunits: $\alpha_2\beta_2$	
Molecular weight of the full protein (Da)	75,336	62,512	
Monomer sequence length (aa)	183	α subunit: 142	β subunit: 147
Monomer sequence (tryptophan W and tyrosine Y highlighted)	MRKIVVAAIAVSLTTVSIT<br>ASASADPSKDSKAQVSA<br>AEAGITGTWYNQLGSTFI<br>VTAGADGALTGTYESAV<br>GNAESRYVLTGRYDSAP<br>ATDGSGTALGWTVAWK<br>NNYRNAHSATTWSGQY<br>VGGAEARINTQWLLTSG<br>TTEANAWKSTLVGHDTF<br>TKVKPSAASIDAAKKAGV<br>NNGNPLDAVQQ	α subunit:<br>MVLSPADKTNVKAAWG<br>KVGAHAGEYGAEALER<br>MFLSFPTTKTYFPHFDLS<br>HGSAQVKGHGKKVADA<br>LTNAVAHVDDMPNALS<br>ALSDLHAHKLRVDPVNF<br>KLLSHCLLVTLAAHLPAEF<br>TPAVHASLDKFLASVSTV<br>LTSKYR	β subunit:<br>MVHLTPEEKSAVTALWG<br>KVNVDEVGGEALGRLLV<br>VYPWTQRFFESFGDLST<br>PDAVMGNPKVKAHGKK<br>VLGAFSDGLAHLDNLKG<br>TFATLSELHCDKLHVDPE<br>NFRLLGNVLVCVLAHHF<br>GKEFTPPVQAAYQKVVA<br>GVANALAHKYH
Tryptophan count per monomer	6	1	2
Tyrosine count per monomer	6	3	3
Total tryptophan residues per protein	24	6	
Total tyrosine residues per protein	24	12	
Extinction coefficient ε at 280 nm (M^{-1} cm^{-1})	169,360	57,630	

Supplementary references

(1) Baibakov, M.; Barulin, A.; Roy, P.; Claude, J.-B.; Patra, S.; Wenger, J. Zero-Mode Waveguides Can Be Made Better: Fluorescence Enhancement with Rectangular Aluminum Nanoapertures from the Visible to the Deep Ultraviolet. *Nanoscale Adv.* **2020**, *2*, 4153–4160.

(2) Chen, R. F. Measurements of Absolute Values in Biochemical Fluorescence Spectroscopy. *J. Res. Natl. Bur. Stand. Sect. Phys. Chem.* **1972**, *76A*, 593–606.

(3) Barnes, W. L.; Horsley, S. A. R.; Vos, W. L. Classical Antennae, Quantum Emitters, and Densities of Optical States. *J. Opt.* **2020**, *22*, 073501.

(4) Regmi, R.; Al Balushi, A. A.; Rigneault, H.; Gordon, R.; Wenger, J. Nanoscale Volume Confinement and Fluorescence Enhancement with Double Nanohole Aperture. *Sci. Rep.* **2015**, *5*, 15852.

(5) Aouani, H.; Mahboub, O.; Bonod, N.; Devaux, E.; Popov, E.; Rigneault, H.; Ebbesen, T. W.; Wenger, J. Bright Unidirectional Fluorescence Emission of Molecules in a Nanoaperture with Plasmonic Corrugations. *Nano Lett.* **2011**, *11*, 637–644.

(6) Bidault, S.; Devilez, A.; Maillard, V.; Lermusiaux, L.; Guigner, J.-M.; Bonod, N.; Wenger, J. Picosecond Lifetimes with High Quantum Yields from Single-Photon-Emitting Colloidal Nanostructures at Room Temperature. *ACS Nano* **2016**, *10*, 4806–4815.

(7) Regmi, R.; Berthelot, J.; Winkler, P. M.; Mivelle, M.; Proust, J.; Bedu, F.; Ozerov, I.; Begou, T.; Lumeau, J.; Rigneault, H.; García-Parajó, M. F.; Bidault, S.; Wenger, J.; Bonod, N. All-Dielectric Silicon Nanogap Antennas To Enhance the Fluorescence of Single Molecules. *Nano Lett.* **2016**, *16*, 5143–5151.

(8) Flauraud, V.; Regmi, R.; Winkler, P. M.; Alexander, D. T. L.; Rigneault, H.; van Hulst, N. F.; García-Parajo, M. F.; Wenger, J.; Brugger, J. In-Plane Plasmonic Antenna Arrays with Surface Nanogaps for Giant Fluorescence Enhancement. *Nano Lett.* **2017**, *17*, 1703–1710.

(9) Bharadwaj, P.; Deutsch, B.; Novotny, L. Optical Antennas. *Adv. Opt. Photonics* **2009**, *1*, 438–483.

(10) Tiwari, S.; Roy, P.; Claude, J.-B.; Wenger, J. Achieving High Temporal Resolution in Single-Molecule Fluorescence Techniques Using Plasmonic Nanoantennas. *Adv. Opt. Mater.* **2023**, *11*, 2300168.

(11) Barulin, A.; Claude, J.-B.; Patra, S.; Moreau, A.; Lumeau, J.; Wenger, J. Preventing Aluminum Photocorrosion for Ultraviolet Plasmonics. *J. Phys. Chem. Lett.* **2019**, *10*, 5700–5707.

(12) Renard, D.; Tian, S.; Ahmadivand, A.; DeSantis, C. J.; Clark, B. D.; Nordlander, P.; Halas, N. J. Polydopamine-Stabilized Aluminum Nanocrystals: Aqueous Stability and Benzo[a]Pyrene Detection. *ACS Nano* **2019**, *13*, 3117–3124.

(13) Roy, P.; Badie, C.; Claude, J.-B.; Barulin, A.; Moreau, A.; Lumeau, J.; Abbarchi, M.; Santinacci, L.; Wenger, J. Preventing Corrosion of Aluminum Metal with Nanometer-Thick Films of Al2O3 Capped with TiO2 for Ultraviolet Plasmonics. *ACS Appl. Nano Mater.* **2021**, *4*, 7199–7205.

(14) Renard, D.; Tian, S.; Lou, M.; Neumann, O.; Yang, J.; Bayles, A.; Solti, D.; Nordlander, P.; Halas, N. J. UV-Resonant Al Nanocrystals: Synthesis, Silica Coating, and Broadband Photothermal Response. *Nano Lett.* **2021**, *21*, 536–542.
